# Integrated transcriptomic landscape of medulloblastoma and ependymoma reveals novel tumor subtype-specific biology

**DOI:** 10.1093/neuonc/noaf251

**Published:** 2025-10-24

**Authors:** Sonali Arora, Nicholas Nuechterlein, Matt Jensen, Gregory Glatzer, Philipp Sievers, Srinidhi Varadharajan, Andrey Korshunov, Felix Sahm, Stephen C Mack, Michael D Taylor, Taranjit S Gujral, Eric C Holland

**Affiliations:** Human Biology Division, Fred Hutchinson Cancer Center, Seattle, Washington (S.A., M.J., G.G., T.S.G., E.C.H.); Department of Pathology, University of Michigan, Ann Arbor, Michigan (N.N.); Human Biology Division, Fred Hutchinson Cancer Center, Seattle, Washington (S.A., M.J., G.G., T.S.G., E.C.H.); Human Biology Division, Fred Hutchinson Cancer Center, Seattle, Washington (S.A., M.J., G.G., T.S.G., E.C.H.); Department of Neuropathology, University Hospital Heidelberg, and CCU Neuropathology, German Consortium for Translational Cancer Research (DKTK), German Cancer Research Center (DKFZ), ­Heidelberg, Germany (P.S., A.K., F.S.); Developmental Neurobiology Department, Neurobiology and Brain Tumor Program, St Jude Children’s Research Hospital, Memphis, Tennessee (S.V., S.C.M.); Department of Neuropathology, University Hospital Heidelberg, and CCU Neuropathology, German Consortium for Translational Cancer Research (DKTK), German Cancer Research Center (DKFZ), ­Heidelberg, Germany (P.S., A.K., F.S.); Department of Neuropathology, University Hospital Heidelberg, and CCU Neuropathology, German Consortium for Translational Cancer Research (DKTK), German Cancer Research Center (DKFZ), ­Heidelberg, Germany (P.S., A.K., F.S.); Developmental Neurobiology Department, Neurobiology and Brain Tumor Program, St Jude Children’s Research Hospital, Memphis, Tennessee (S.V., S.C.M.); Neuro-oncology Research Program, Department of Pediatrics, Section of Hematology-Oncology, Baylor College of Medicine, Houston, Texas (M.D.T.); Human Biology Division, Fred Hutchinson Cancer Center, Seattle, Washington (S.A., M.J., G.G., T.S.G., E.C.H.); Human Biology Division, Fred Hutchinson Cancer Center, Seattle, Washington (S.A., M.J., G.G., T.S.G., E.C.H.)

**Keywords:** copy number, gene fusions, pathways, RNA-Seq, subtyping

## Abstract

**Background:**

Medulloblastoma and ependymoma are common pediatric central nervous system tumors with significant molecular and clinical heterogeneity. While molecular subgrouping has enabled classification into molecular subtypes, the extent of heterogeneity within these subgroups remains poorly defined.

**Methods:**

We collected bulk RNA sequencing data from 888 medulloblastoma and 370 ependymoma tumors to establish a comprehensive reference landscape. After rigorous batch effect correction, normalization, and dimensionality reduction, we generated a unified landscape to explore gene expression, signaling pathways, RNA fusions, and copy number variations.

**Results:**

Our transcriptional analysis revealed distinct clustering patterns, including two primary ependymoma compartments, EPN-E1 and EPN-E2, each with specific RNA fusions and molecular signatures. In medulloblastoma, we observed precise stratification of Group 3/4 tumors by subtype and in Sonic Hedgehog (SHH) tumors by patient age. We also identified subtype-specific pathways and gene fusions, enriched in each group.

**Conclusions:**

This transcriptomic landscape serves as a resource for biomarker discovery, diagnostic refinement, and prediction of tumor biology and outcome. By enabling projection of new patients’ bulk RNA-seq data onto the reference map using nearest neighbor analysis, the framework supports accurate subtype classification. The landscape is publicly available via Oncoscape, an interactive platform for global exploration and application.

## Graphical abstract

**Graphical Abstract:**
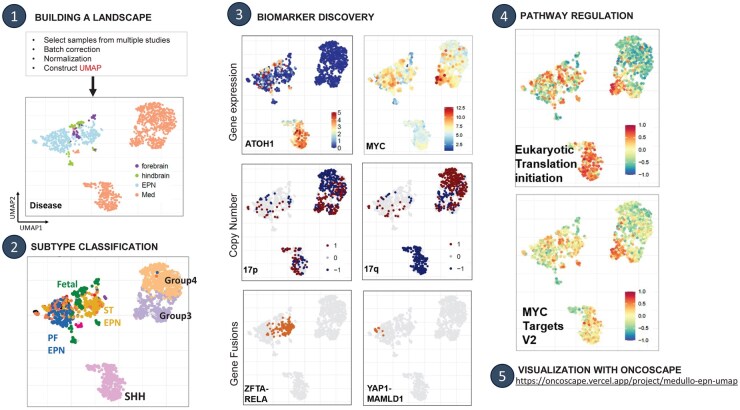

Key Points

- Interactive landscape to visualize gene expression, pathway regulation, copy number and RNA gene fusions, built from a large cohort of publicly available medulloblastoma and ependymomas.
- Distinct pathways, gene fusions are identified for different medulloblastoma and ependymoma subtypes using large cohort of bulk RNASeq data.
- Consensus clustering using bulk RNASeq data identifies identical subtype to subtypes identified by multi-omic analysis.

Importance of the Study

This study presents the largest uniformly processed bulk RNA-Seq analysis of pediatric medulloblastoma and ependymoma to date, integrating data from 1258 tumors. Using rigorous normalization and clustering methods, we define transcriptomic landscapes that reveal novel subtype-specific pathways and gene fusion patterns. Importantly, these findings from medulloblastoma are validated using single-cell RNA-Seq data from 25 patients, confirming that bulk RNA-derived signals reflect tumor cell-intrinsic biology. Our interactive framework enables real-time mapping of new tumors, with translational potential for improving subtype classification, biomarker discovery, and outcome prediction. Unlike prior studies focused on fixed subtypes or small cohorts, our approach accommodates molecular heterogeneity and offers a scalable, clinically applicable tool for the neuro-oncology community. The integration into Oncoscape allows global access to a user-friendly diagnostic interface. Together, this work provides a robust, reproducible foundation for translational studies and clinical implementation of RNA-based tumor stratification.

Medulloblastoma, a highly malignant brain tumor of the cerebellum, is the most common pediatric central nervous system (CNS) cancer, accounting for ∼20% of childhood brain tumors.^1^ Once considered a single entity, medulloblastoma is now recognized to comprise four molecular subtypes per the WHO 2021 classification: Wingless (WNT), Sonic Hedgehog (SHH), Group 3, and Group 4.^2^ These ­subtypes differ in molecular drivers, clinical behavior, prognosis, and treatment response. Advances in genomic technologies have uncovered diverse mutations, copy number alterations (CNA), and epigenetic changes across these ­subtypes, offering new insights and potential targets for precision therapy.

Ependymomas (EPNs) arise from neuroepithelial cells and occur throughout the CNS across all ages. In children, they comprise ∼10% of malignant CNS tumors, with ∼30% diagnosed under age three.^3^ Molecular profiling has revealed distinct subtypes with unique clinical and pathological features. Supratentorial ependymomas are primarily driven by gene fusions involving either *ZFTA* (formerly *C11orf95*, often fused to *RELA*) or *YAP1.*^4^^,^^5^ Posterior fossa ependymomas are now stratified into PF-A and PF-B subtypes, with an additional NEC/NOS classification. These distinctions have critical diagnostic and prognostic implications, emphasizing the importance of molecular characterization in clinical settings.

In this study, we present a unified transcriptomic landscape of medulloblastoma and ependymoma using harmonized bulk RNA-seq data from five medulloblastoma and eight ependymoma studies.^6-9^ Following batch correction and normalization, we used dimensionality reduction to construct a reference landscape integrating 888 medulloblastoma and 370 ependymoma tumors. Compared to a previous multi-disease visualization tool that included ­limited CBTN-derived samples (93 medulloblastomas and 117 ependymomas),^10^ our work represents the largest collection of bulk RNA-seq data for these tumors to date.

This landscape enables detailed interrogation of gene expression patterns, signaling pathways, gene fusions, and copy number profiles across molecular subtypes. It recapitulates known classifications while offering insights into intra-subtype heterogeneity and developmental parallels. It also supports retrospective quality control and clinical reassessment of misdiagnosed or ambiguous cases.

Importantly, we incorporated fetal brain reference samples to better contextualize tumor expression profiles within CNS developmental trajectories. This developmental anchoring aids in interpreting tumor cell-of-origin features and could inform age-specific biology and therapy response.

Finally, we have made this resource publicly accessible via Oncoscape^11^ (https://oncoscape.vercel.app/project/medullo-epn-umap), providing an interactive platform for clinicians and researchers to explore genes, discover biomarkers, and guide translational research in pediatric neuro-oncology.

## Materials and methods

### Collection of publicly available RNA sequencing data

Raw RNA-seq data for medulloblastoma and ependymoma were retrieved from public repositories (Table S1). The ­Heidelberg dataset was obtained from the Department of Neuropathology, University Hospital Heidelberg.

### RNA-Seq data processing and visualization

Raw reads were assessed using FastQC (v0.11.9) and summarized with MultiQC (v1.9). Reads were aligned to the Gencode GRCh38.primary_assembly reference genome using STAR^12^ (v2.7.7), and gene-level counts were generated with HTSeq^13^ (v0.11.0) using Gencode^14^ V39 primary assembly annotations. Gene counts from all datasets were aggregated, and batch effects were corrected with ComBat-seq (“sva”).^15^ Variance-stabilized expression values (VST) were computed using “DESeq2.”^16^

### Clustering approaches

Uniform manifold approximation and projection (UMAP) was applied to VST-normalized protein-coding gene expression to generate medulloblastoma and ependymoma reference maps using the **umap** R package. Parameters: n_neighbors = 15, metric = “euclidean,” n_epochs = 200, min_dist = 0.5, and n_components = 2 (2D) or 3 (3D for Oncoscape). consensusClusteringPlus^17^ was used to determine the optimum k for SHH and Group 3/group 4 medulloblastoma and for ependymoma samples. WNT medulloblastoma and fetal samples were excluded during ependymoma subclustering.

### Gene fusion detection from RNA-Seq

Gene fusions were identified using Arriba^18^ (v2.1.0) and STAR-Fusion^19^ on STAR two-pass aligned RNA-Seq data. Only high-confidence fusions identified by both tools and involving at least one protein-coding gene (GENCODE v39, GRCh38.p14) were retained.

### Copy number alterations detection from RNA-Seq

Large-scale CNAs, including arm-level events, were inferred using CaSpER^20^ on bulk RNA-Seq data. BAFExtract (https://github.com/akdess/) provided source code, genome list, and pileups. Cytoband and centromere data for hg38 were obtained from UCSC Genome Browser.

### Kaplan-Meier survival analysis

Kaplan-Meier curves were generated using samples with known recurrence status and time to recurrence or last follow-up. *P*-values were computed using the **survival** R package (v3.5.7).

For pathway-level analysis, gene set variation analysis (GSVA) scores (−1 to 1) were dichotomized at 0.6 (High > 0.6, Low ≤ 0.6) and evaluated using log-rank tests and multivariable Cox models adjusted for age, sex, and cluster (C5 reference).

### Differential gene expression analysis

Differential expression analysis was performed using DESeq2.^16^ Significantly regulated genes in each comparison were identified based on FDR (< 0.05) and log 2-fold change (> 0.3) or fold change of 25%.

### Gene set variation analysis pathway analysis

Pathway gene sets from KEGG,^21^ Biocarta,^22^ Reactome,^23^ and GO Biological Processes were obtained from MSigDB v7.2 (https://www.gsea-msigdb.org/gsea/msigdb/collections.jsp). Gene set variation analysis^24^ was applied to batch-corrected VST counts, generating scores (−1 to 1) for each sample, which were visualized using ggplot2.^25^

### Placing new patients on UMAP reference map

Variance stabilizing transformation counts from 12 medulloblastoma NOS samples were used as test data; all other samples served as training data. We applied the k-nearest neighbors–based method from Thirimane et al,^26^ to project test samples onto the reference UMAP using their nearest neighbors. The resulting UMAP coordinates were appended to the existing UMAP object and visualized in R with ggplot2.

### Oncoscape integration

Expression and clinical matrices were converted to cBioPortal-compatible formats for upload into Oncoscape. Custom color schemes and predefined views matching ­manuscript figures were stored in a JSON-formatted Oncoscape updates.txt file (See upload documentation at https://github.com/FredHutch/OncoscapeV3/blob/master/docs/upload.md.)

## Results

### Constructing a reference landscape for medulloblastoma and ependymoma

We gathered 370 ependymoma samples and 888 medulloblastoma samples from North America and Europe to construct a comprehensive reference landscape for both tumor types. The ependymoma cohort^4^^,^  ^27-30^ included 134 supratentorial, 135 posterior fossa, 77 ependymoma (NOS), 11 anaplastic, 9 myxopapillary, and 4 spinal ependymoma samples, sourced from across North America and Europe. The medulloblastoma cohort^7^^,^  ^31^ consisted of 364 Group 4, 229 Group 3, 274 SHH, 9 WNT, and 12 medulloblastoma (NOS) samples, all collected from North America. Additionally, we incorporated 100 healthy brain samples at various stages during embryonic and post-natal development^32^ to serve as a control dataset. These control samples comprised 48 forebrain and 52 hindbrain samples, covering a broad developmental range: 65 samples were from 4 to 19 weeks post-conception and 35 post-natal samples (Figure S1, Table S1).

Raw sequencing reads from each sample were aligned to the human genome reference hg38, and gene counts were obtained for each gene. Focusing on protein-coding genes, we corrected for batch effects using the ComBatSeq function from the R package “sva.” Gene expression data was then normalized using variance stabilizing transformation (VST). To ensure the robustness of the clustering, we performed a comprehensive comparison of Principal component analysis (PCA), t-distributed stochastic neighbor embedding (t-SNE), and UMAP with and without batch correction and explored various normalization methods **(**Figure S1a-i).^10^^,^  ^26^

**Figure 1.**
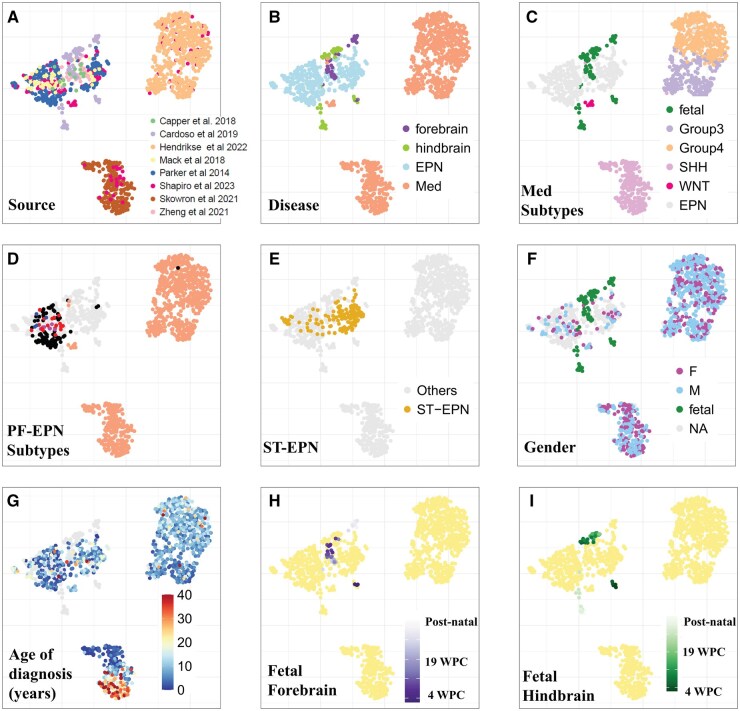
Generation of the medulloblastoma and ependymoma landscape with clinical and genomic metadata. (A) UMAP visualization colored by dataset source. (B) UMAP colored by disease type. (C) UMAP colored by subtypes for both medulloblastoma and ependymoma. (D) UMAP colored by subtypes within the posterior fossa. (E) UMAP highlighting supratentorial ependymomas (orange), with all other samples shown in grey. (F) UMAP colored by gender where available: Female (pink), Male (blue), and fetal samples (green). (G) UMAP colored by patient age at the time of tumor sample acquisition. (H) UMAP colored by age of forebrain samples. (I) UMAP colored by age of hindbrain samples.

After overlaying known biological information, we selected the VST-normalized UMAP as our final reference landscape because it demonstrated no batch effects based on data source (Figure 1A) and effectively captured clusters corresponding to publicly known subtypes of the disease (Figure 1B).

### Overall structure of the reference landscape

Consistent with published analyses,^3^ the medulloblastoma samples formed four distinct clusters corresponding to the SHH, Group 3, and Group 4 subtypes. Group 3 and Group 4 medulloblastomas were positioned along a continuum, consistent with previous reports. Notably, the nine WNT medulloblastoma samples clustered with the ependymoma samples, distinctly separate from the other medulloblastoma subtypes (Figure 1C). The ependymoma clusters and the WNT medulloblastomas clustered closely with the developmental brain samples (Figure 1C).

Coloring in the landscape based on the metadata collected for each sample, the ependymoma samples appeared to be divided into two major groups: posterior fossa ependymomas were predominantly localized in one cluster of the UMAP, while supratentorial ependymomas were primarily situated in the other (Figure 1D and E). These two clusters corresponded to EPN-E1 and EPN-E2.^33^ For samples where data were available, we further colored the posterior fossa ependymoma samples by annotated subtypes. The 17 PF-A samples were distributed across E2, the 4 PF-B samples formed a tight cluster at the top, and the 9 PF-SE (subependymoma) samples clustered in the middle of the posterior fossa region (Figure 1D).

Overlaying sex information on our UMAP revealed no distinct regional patterns separating male and female samples on the reference landscape (Figure 1F). However, when coloring the UMAP by the age of tumor samples, a clear age-based pattern emerged within the SHH medulloblastoma cluster. Specifically, older patients’ samples predominantly occupied one specific region of the cluster, while younger patients’ samples concentrated in the upper half, further validating our UMAP's ability to differentiate between previously reported SHH medulloblastoma subgroups (­Figure 1G). Additionally, we visualized the age distribution for the forebrain and hindbrain samples (Figure 1H and I) and noted that the early fetal forebrain and hindbrain samples clustered closely with the supratentorial ependymomas on the right side.

### Mapping medulloblastoma genomic features onto the UMAP landscape

While UMAP visualizations provide an intuitive representation of the data, these clusters are grounded in rigorous analyses performed in the original high-dimensional space and are further supported by biological validation using well-established genomic and transcriptomic markers from the literature. By Integrating known clinical features into the UMAP, distinct regionalization of different subtypes is revealed. We employed three methods: Arriba^18^ and STAR-Fusion^19^ to detect RNA fusions and CaSpER^20^ to infer copy number patterns for each sample in our dataset.

**Figure 2.**
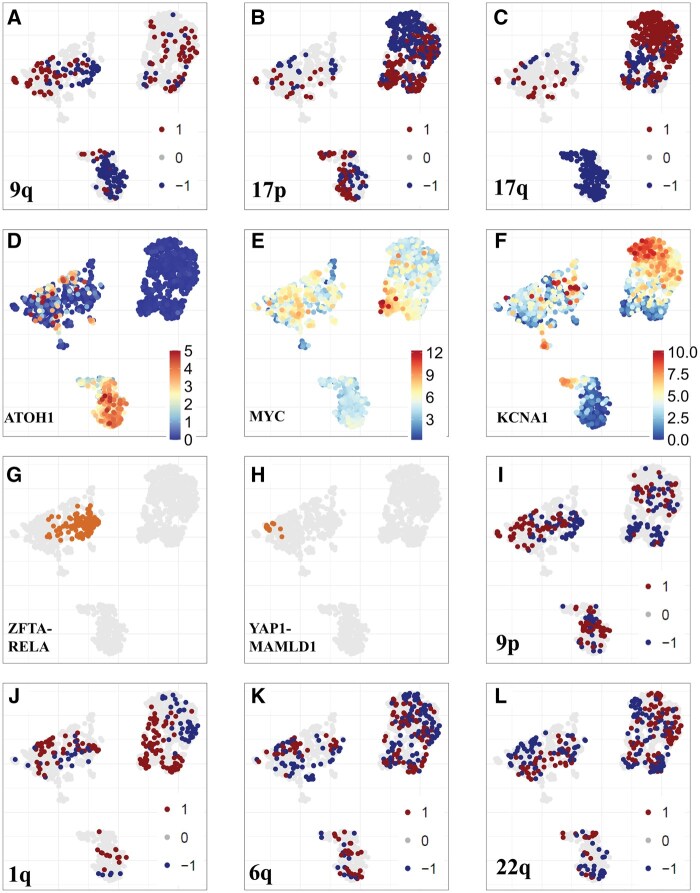
Validation of the reference landscape through copy number, gene fusions, and gene expression patterns. (A-C) UMAP colored by copy number alterations to validate medulloblastoma subtypes: (A) 9q, (B) 17p, (C) 17q (red for gains, blue for deletions). (D-F) UMAP colored by gene expression levels: (D) ATOH1, (E) MYC, (F) KCNA1. (G and H) UMAP colored by gene fusions to confirm ependymoma subtypes: (G) ZFTA-RELA, (H) YAP-MAMLD1. (I-L) UMAP colored by copy number patterns in ependymomas: (I) 9p, (J) 1q, (K) 6q, (L) 22q.

For medulloblastomas, it is known that the SHH (Sonic Hedgehog) subgroup is marked by significant deletions on chromosome 9q,^7^ while Group 3 and Group 4 medulloblastomas are associated with a loss of chromosome 17p and a gain of 17q.^6^^,^^34^ In our cohort, 32.12% (88/274) of SHH medulloblastomas exhibited a loss of chr9q (Figure 2A), 17.90% (41/229) of Group 3 medulloblastomas had a loss of chr17p, and 43.95% (160/364) of Group 4 medulloblastomas exhibited this loss (Figure 2B). Furthermore, 62.08% (226/364) of Group 4 medulloblastomas and 22.70% (52/229) of Group 3 medulloblastomas had a gain of chr17q (Figure 2C, ­Figure S1J).

When we overlaid gene expression patterns onto the reference landscape, we observed distinct differences between SHH medulloblastomas and other tumor types. Sonic Hedgehog medulloblastomas exhibited high expression of ATOH1 (Figure 2D, Figure S1K), SFRP1 and HHIP (Figure S2a and b), consistent with prior reports highlighting their elevated expression in this subtype.^35^ In contrast, Group 3 medulloblastomas were characterized by elevated expression of MYC (Figure 2E), GABRA5, and IMPG2 (Figure S2C and D), while Group 4 medulloblastomas showed high expression of KCNA1 (Figure 2F), EOMES, and RBM24 (­Figure S2E and F), corroborating existing literature that associates these genes with this subtype.

Consistent with previous studies, we identified several reported gene fusions across various medulloblastoma subtypes.^35^ Specifically, as described by Luo et al, in our analysis 14.23% (39/274) of SHH medulloblastomas exhibited fusions involving CCDC196: LINC02290 (Figure S2G). In contrast, among Group 3 and Group 4 medulloblastomas, 27% (62/229) of group 3 and 54% (200/364) of group 4 showed gene fusion in GJE: VTA1, 6.9% (16/229) showed gene fusion in PVT1: PCAT1, 4.8%(11/229) of group 3 and 6.9% (25/364) of group 4 showed gene fusion in TUBB2B: LMAN2L and 28/364(7.69%) of group 4 in ELP4: IMMP1L (Figure S2H-K).

### Mapping ependymoma genomic feature onto the UMAP landscape

Supratentorial ependymomas (ST-EPNs) are frequently defined by specific gene fusions, most notably ZFTA-RELA^36^ and YAP1 fusions,^5^ as well as recurrent losses of the entire chromosome 9 arm.^5^ In our study, we observed that 71.64% (96/134) of ST-EPNs harbored the ZFTA-RELA fusion (Figure 2G), while 6.71% (9/134) exhibited YAP1-MAMLD1 fusions. Notably, ZFTA-RELA fusions localized to a specific region on the UMAP, whereas YAP1-MAMLD1 fusions clustered on the opposite side, aligning more closely with posterior fossa ependymoma samples (Figure 2H). Additionally, 26.86% (36/134) of ST-EPN tumors showed deletions in the short arm of chromosome 9 (chr9p), and 23.88% (32/134) had deletions in the long arm (chr9q) (Figure 2A and I).

By contrast, posterior fossa ependymomas are typically characterized by a gain of chromosome 1q^37^ and losses of chromosomes 6q and 22q.^5^ Within our cohort, 12.59% (17/135) of posterior fossa ependymomas exhibited a 1q gain, 12.2% (17/134) had a 6q loss, and 11% (16/134) had a 22q loss (Figure 2J-L). As previously reported, posterior fossa ependymomas exhibited elevated expression of WNT5A^37^ (Figure S2L), TGFB1^37^ (Figure S2M), and HOXB2^38^ (Figure S2N), consistent with their established molecular signature. Similarly, supratentorial ependymomas demonstrated high expression of IGF2^39^ (Figure S2O), L1CAM^40^ (Figure S2P), and CCND1^41^ (Figure S2Q), corroborating findings from earlier studies that identified these genes as key markers for this subtype. These results validate and extend existing knowledge of the gene expression profiles specific to ependymoma subtypes.

### Consensus clustering delineates stable molecular compartments in medulloblastoma

To define the transcriptional substructure of pediatric brain tumors, we performed consensus clustering on the full gene expression matrix, rather than the UMAP embedding. Unlike clustering in low-dimensional spaces like UMAP—which can distort distances and obscure subtle transcriptional differences—consensus clustering on the complete expression dataset provides a more stable and biologically meaningful stratification by assessing the reproducibility of sample groupings across multiple resampling iterations.

**Figure 3.**
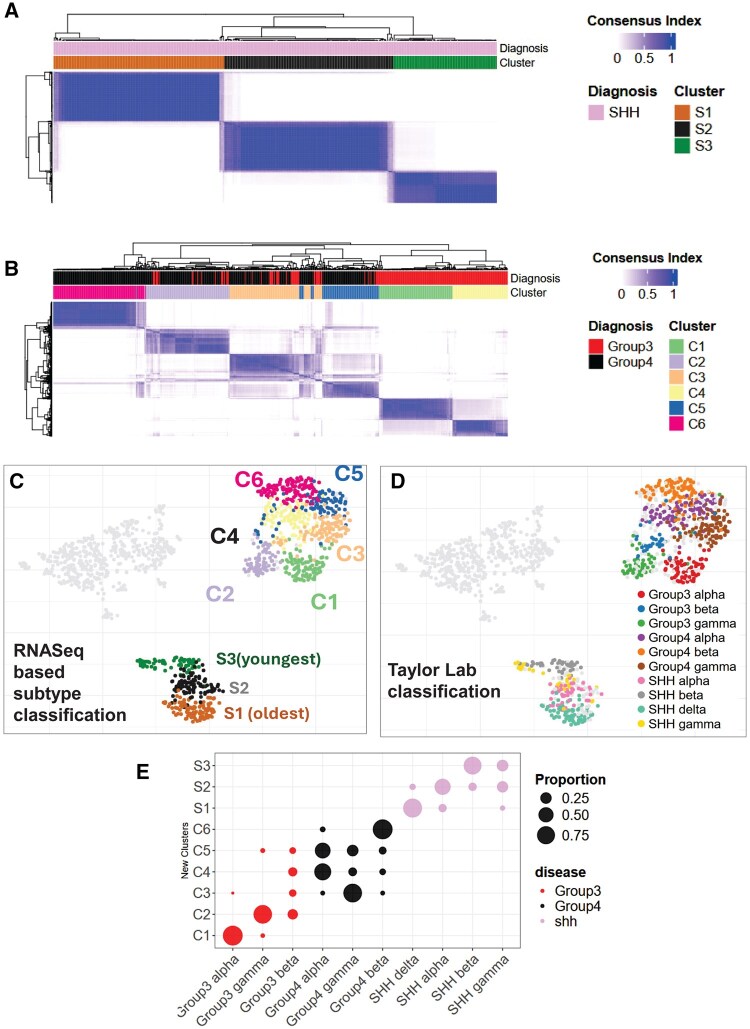
Consensus clustering reveals distinct subgroups of medulloblastoma. (A) Consensus clustering of SHH medulloblastoma reveals three distinct clusters for SHH medulloblastoma, and (B) six different subclusters for group 3 and group 4 medulloblastoma (D) UMAP colored by subtype as obtained from consensus clustering (D) UMAP colored by subtype classification according to Cavalli et al. (D) Dotplot showing the proportion of samples shared in Taylor Lab classification and RNASeq based consensus subtype classification.

Using this approach, SHH medulloblastomas resolved into three distinct subclusters (Figure 3A), aligning closely with previously described age-dependent subtypes. Group 3/4 medulloblastomas exhibited substantial heterogeneity, separating into six expression-based clusters (Figure 3B and C), indicating a finer resolution of transcriptional diversity than previously reported.

To benchmark our RNA-seq–based clustering, we compared it to the multi-omic classification described by Cavalli et al. (Figure 3D). We found strong concordance between our clusters and the subtypes defined by Cavalli et al and Skowron et al, with substantial sample overlap (Figure 3E). Notably, our method—using only RNA-seq data—achieved comparable stratification of SHH medulloblastomas, accurately distinguishing S1 (SHHδ), S2 (SHHα), and S3 (SHHβ/γ) subgroups. These corresponded to median ages of 25, 6.3, and 1.6 years, respectively, highlighting the age-associated structure captured by our clustering.

In addition to transcriptional resolution, our approach revealed clinically relevant associations. Patients in S2 with 9q deletions exhibited significantly improved survival ­compared to copy-neutral cases (*P* = .0029, Figure S3A). As previously reported by Cavalli et al., S2 also showed enrichment for 9p amplifications, 9q deletions, and 10q deletions, while S1 exhibited a higher frequency of 14q deletions (­Figure S3B, Table S2). These genomic features reinforce the robustness of our RNA-based subtyping.

Group 3/4 tumors were similarly stratified into six distinct clusters. Group 3 tumors split into C1, C2, and C4, while Group 4 tumors segregated into C3, C5, and C6. Consistent with Cavalli et al., C2 showed strong MYC upregulation and frequent chromosome 8 gains (8p: 52.77%, 8q: 63.88%; Figure 2E, Figure S3C, Table S2). By contrast, C1 was marked by gain of chromosome 7 (7p: 25%, 7q: 37.5%), loss of chromosome 8 (8p: 39.58%, 8q: 31.25%), and gain of 14q (52.08%) (Table S2). Subgroup C3 exhibited widespread loss of both 8p (68.46%) and 8q (63.06%) (Table S2).

Further, we also noted a subgroup of C4 demonstrated significant sex-based survival differences, with males faring worse than females (*P* = .015, Figure S3D). Additionally, C4 patients with deletions in chromosome 4p had better outcomes than those who were copy-neutral for this region (*P* = .032, Figure S3E, Table S2). Isochromosome 17 also displayed varied copy number patterns across clusters: 17q gain was seen in C1 (21.87%), C2 (23.6%), C3 (47.47%), C4 (46.36%), C5 (52.94%), and C6 (76.47%), while 17p loss was most prominent in C1 (16.6%) (Table S2).

### Bulk and single-cell RNA-Seq concordantly define subtype-specific expression patterns and pathways in medulloblastoma

To uncover biologically distinct signaling programs within medulloblastoma, we GSVA on RNA-seq data from each molecular subtype. This revealed unique transcriptional signatures and signaling pathway enrichments distinguishing SHH, Group 3, and Group 4 tumors.

**Figure 4.**
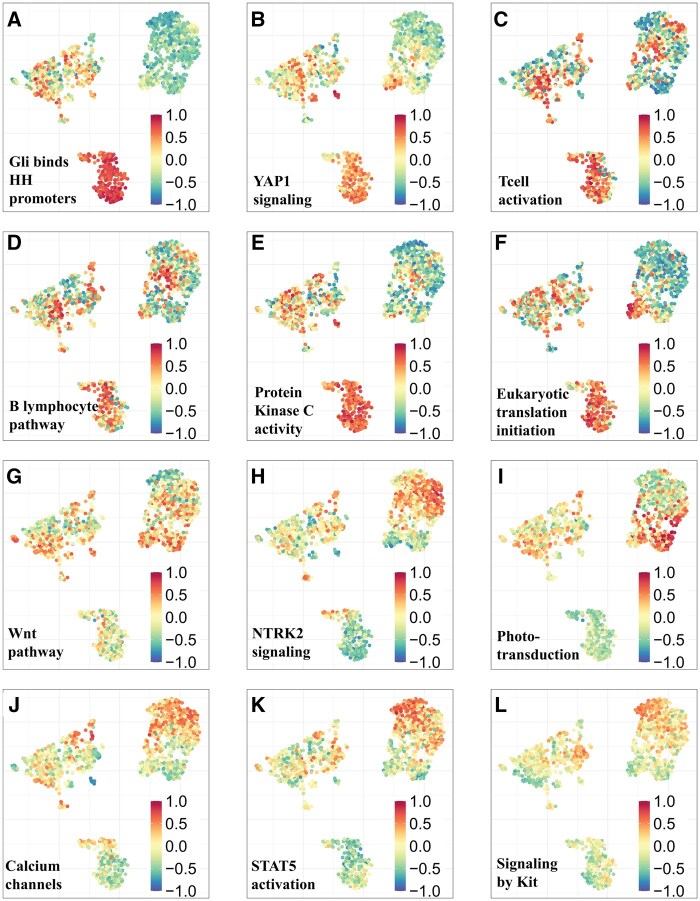
Group 3 and Group 4 Medulloblastoma Subtypes with Distinct Molecular Profiles Identified by RNA-Seq. (A-F) Pathways upregulated in SHH medulloblastoma compared to Group 3 and Group 4 medulloblastoma samples (G-L). UMAP colored in by GSVA score, A score closer to 1 denotes up-regulation of pathway, and closer to -1 denotes down-regulation of pathway.

Across all SHH tumors, we identified distinct pathways that were uniquely upregulated in this subtype, distinguishing them from other medulloblastoma subtypes. Specifically, pathways involving Gli protein binding to promoters, RUNX3 regulation of YAP1-mediated transcription, ribosome-related processes, and B-lymphocyte signaling were prominently upregulated in SHH tumors. We also noted additional upregulated pathways, such as protein kinase C activity, metabolic processes, wound healing, nonsense mediated decay, and T-cell activation (Figure 4A-F, Figure S3F).

The Group 4 subgroups (C3, C5, and C6) displayed distinct pathway regulation compared to the Group 3 subgroups. Specifically, the MYC-amplified Group 3 subgroup C2 was enriched for pathways related to translation, Wnt signaling, phototransduction activation, Telomerase Reverse Trans­criptase (TERT) pathway, and voltage-gated channels (­Figure 4G-L, Figure S4A-E). In contrast, Group 4 subgroups showed upregulation in pathways such as NTRK2 signaling, STAT5 activation, signaling by leptin, IL22BP pathway, KIT signaling, and presynaptic depolarization and calcium signaling (Figure 4G-L, Figure S4H-L).

To validate the novel pathways identified in each subtype, we analyzed single-cell RNA sequencing (scRNA-seq) data from 25 patients, comprising 5 WNT, 3 SHH, 8 Group 3, and 9 Group 4 samples, obtained from Hovestadt et al. Following data preprocessing using Seurat, we observed that WNT and SHH subtypes formed distinct clusters, whereas Group 3 and Group 4 samples exhibited closer clustering patterns (Figure S4J). This spatial relationship mirrors the clustering observed in the UMAP projection generated from bulk RNA-seq data. Differential gene expression analysis further corroborated subtype-specific patterns consistent across both scRNA-seq and bulk RNA-seq datasets. For instance, SHH samples exhibited elevated expression of ATOH1, SFRP1, and HHIP, while Group 3 displayed overexpression of MYC, GABRA5, and IMPG2, and Group 4 demonstrated higher expression of KCNA1, EOMES, and RBM24 (Figure S4K, S4L). Gene set variation analysis reinforced the activation of novel pathways identified in scRNA-seq data, aligning with findings from bulk RNA-seq. Notably, NTRK2 signaling was most prominent in Group 4, and WNT signaling was enriched in Group 3, among other pathway-specific enrichments (Figure S4M).

### Clustering reveals two distinct ependymoma subgroups with distinct gene fusions

To validate and build upon prior molecular subtype classifications, we reanalyzed the same ependymoma samples previously studied by Chan et al^33^ who identified two robust consensus clusters—EPN-E1 and EPN-E2—using bulk RNA-seq data.

**Figure 5.**
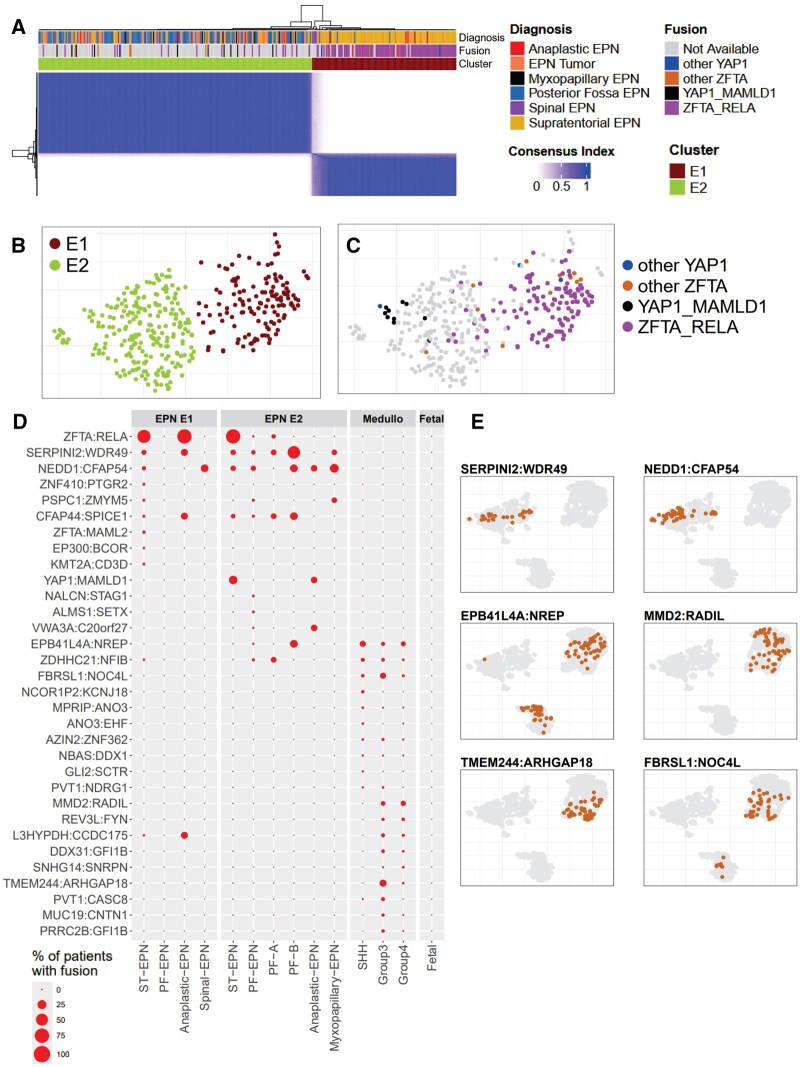
Ependymoma segregate into two clusters EPN-E1 and EPN-E2. (A) Consensus clustering of ependymoma reveals two clusters, EPN-E1 and EPN-E2. (B) UMAP colored by subtype EPN-E1 and EPN-E2 as obtained from consensus clustering. (C) UMAP showing the distribution of commonly studied gene fusions in ependymoma: ZFTA-RELA (purple), YAP1-MAMLD1 (black), other YAP1 fusions (blue), and other ZFTA fusions (brown). (D) Dot plots showing the gene fusions and their frequencies in EPN-E1 and EPN-E2, as well as across medulloblastoma subtypes. (E) UMAP colored in by patients displaying gene fusions regionalized in distinct ependymoma and medulloblastoma subtypes.

Reapplying consensus clustering to this dataset, we recapitulated this two-subgroup structure (Figure 5A). To ensure subtype-specific resolution, WNT medulloblastoma and fetal brain samples were excluded from the ependymoma clustering analysis. When visualized using UMAP, the samples colored by EPN-E1 and EPN-E2 assignments formed two clearly separable clusters, confirming the reproducibility of these transcriptional subtypes in our dimensionality-reduced reference landscape (Figure 5B). To rule out the possibility that the observed transcriptomic differences could be due to variations in tumor purity rather than genuine biological differences, we estimated tumor purity and cell composition using the PUREE^42^  *and* ESTIMATE^43^ methods. Neither method showed differences across clusters in tumor purity or stromal infiltration (Figure S5A and B). We observed that the forebrain and hindbrain samples clustered closely to the ependymoma samples, we thus performed a correlation analysis of EPN samples using top8000 most variable genes and observed a strong correlation with early developmental time points, as opposed to later stages (Figure S5C and D).

EPN-E1 was predominantly composed of supratentorial ependymomas (ST-EPNs), comprising 77% of the cluster, whereas EPN-E2 was enriched for posterior fossa ependymomas (PF-EPNs, 55%), ST-EPNs (12%), ependymoma NOS samples (25%), myxopapillary EPNs (3.85%), and Anaplastic EPNs (2.56%). Tumors diagnosed as myxopapillary ependymomas are exclusively localized within or near the EPN-E2 cluster, whereas spinal and anaplastic ependymomas were distributed across both EPN-E1 and EPN-E2 (Figure 5B). We note that while some of the diagnosis reported here may be outdated with respect to the 2021 WHO classification, we have presented the metadata as originally annotated to ensure transparency and consistency.

A subset of ependymomas have recurrent chromosomal translations that generate oncogenic gene fusions,^28^ which have been shown to drive tumorigenesis in mouse models.^36^ We analyzed gene fusion profiles across EPN-E1 and EPN-E2 clusters and observed distinct patterns between them. The EPN-E1 cluster, predominantly composed of ST-EPNs, was enriched for the canonical ZFTA: RELA gene fusion, found in 82% of samples (87/105; Table S3A). Among EPN-E2, 31.0% (9/29) harbored a YAP1: MAMLD1 fusion, and 27.6% (8/29) carried a ZFTA: RELA fusion (Figure 5C, Table S3B). This suggests that while ZFTA: RELA remains a dominant marker in EPN-E1, a portion of ST-EPNs in EPN-E2 also harbor this fusion, indicating that gene fusion status alone is not uniquely causal for generation of the EPN-E1 gene expression pattern.

Further investigation of the ST-EPNs with ZFTA: RELA fusions in EPN-E2 revealed no other recurrent gene fusions (Table S3C). All five anaplastic ependymomas in EPN-E1 carried the ZFTA: RELA fusion (Table S3D), whereas the six anaplastic cases in EPN-E2 showed no ZFTA: RELA or other recurrent fusions (Table S3E).

As previously described, we also identified non-RELA ZFTA fusions in EPN-E1, including ZFTA: NCOA2 (3/105, 2.9%) and ZFTA: MAML2 (3/105, 2.9%) (Figure 5D). By contrast, EPN-E2 showed distinct fusions not typically associated with ST-EPNs, such as NEDD1: CFAP54, observed in 10.1% (13/129) of PF-EPNs (Table S4A, Figure 5E). The small number of PF-EPNs present in EPN-E1 (*n* = 5) did not exhibit any recurrent gene fusions (Table S4B). Due to limited sample sizes, we were unable to identify fusion trends in PF-A (*n* = 17), PF-B (*n* = 4), or myxopapillary ependymomas (*n* = 9) (Tables S4C-E).

By contrast, the medulloblastoma samples exhibited a higher frequency of RNA fusions per sample compared to ependymomas (Figure S5H) and differed markedly from that of the ependymomas, potentially generated by DNA rearrangement gene fusion or RNA trans-splicing mechanisms. While EPBH41L4A: NREP (14%, 5.2% and 8.79% in SHH, Group 3 and group 4 medulloblastoma respectively, Table S5a-c) was found in all three medulloblastoma subtypes, MMD2: RADIL (7.42% and 10.16% respectively), TMEM244: ARGHAP18 (19.65% and 0.82% respectively) and FBRSL1: NOC4L (14.41% and 2.74% respectively) fusions were seen only in grade 3 and grade 4 subtypes (Figure 5E).

### Distinct gene and pathway regulation in EPN-E1 and EPN-E2 subgroups

**Figure 6.**
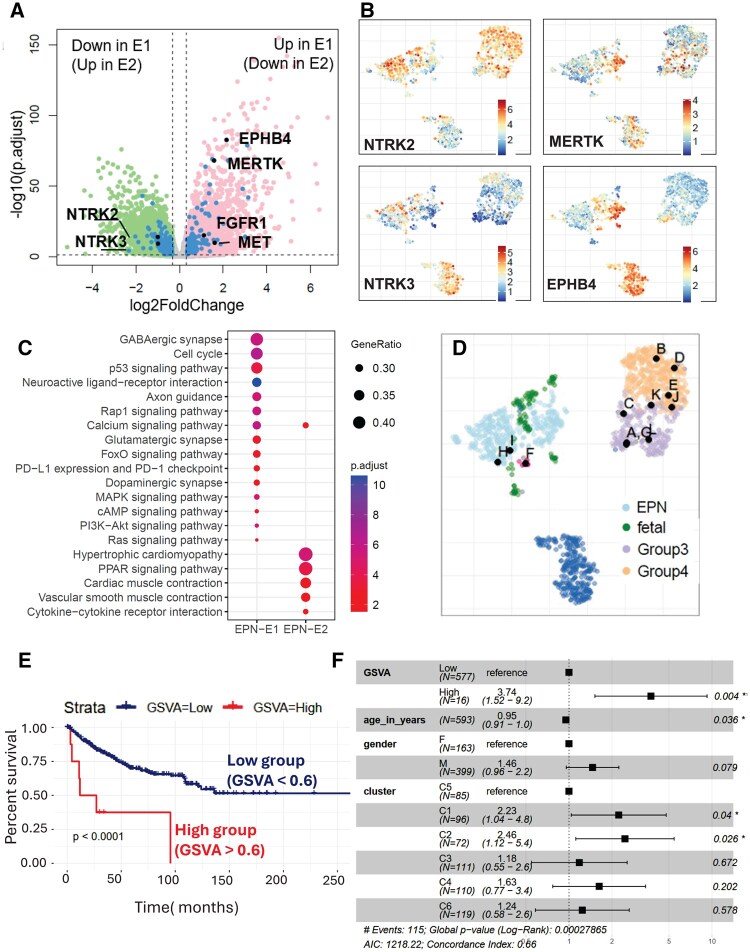
Contrasting Differences Between EPN-E1 and EPN-E2 and integrating new patient data onto reference landscape. (A) Volcano plots showing differentially expressed genes in EPN-E1 (pink) and EPN-E2 (green), with differentially regulated kinases highlighted in blue, the top tyrosine kinase receptors are labeled in black. (B) Gene expression levels of tyrosine receptor kinases: NTRK2, NTRK3, MERTK, and EPHB4. (C) Dot plot illustrating pathways upregulated in EPN-E1 and EPN-E2. (D) Using the k-nearest neighbors algorithm to assign subtypes to 12 NOS medulloblastoma samples. (E) Kaplan-Meier curves for HALLMARK_MYC_TARGETS_V2 (i), dichotomized at the pre-specified GSVA cutoff (High = GSVA > 0.6, Low = GSVA ≤ 0.6). Log-rank *P*-values are shown. (F) Multivariable Cox forest plots for the same pathways, adjusted for age, sex, and cluster (reference C5). Hazard ratios (HR) and 95% CIs are shown for High vs Low (MYC targets: HR 3.74, 95% CI 1.52-9.20, *P* = .004) alongside covariates. Global model metrics: events = 115, c-index ≈ 0.66-0.67.

Differential gene expression analysis between EPN-E1 and EPN-E2 revealed 106 kinases were upregulated in EPN-E1 and another 105 kinases were up-regulated in EPN-E2 (Figure 6A, highlighted in pink). Several tyrosine receptor kinases, such as oncogenic driver MERTK and EPHB4 were up-regulated in EPN-E1 (Figure 6B) and (Table S6a) and NTRK2/3 (Figure 6B) was up-regulated in EPN-E2, which play an oncogenic role in adult glioma^44^ and several other cancer types.^45^ Of note, MerTK was found to be an oncogenic driver in the ZFTA-RELA fusion-driven mouse model of EPN.^33^ The gene expression profiles of E2 ZFTA-RELA tumors are significantly different from the E1 ZFTA-RELA tumors (Figure S6A). Notably, the E1 non-ZFTA-RELA tumors show greater similarity to E1 ZFTA-RELA tumors than to E2 ZFTA-RELA tumors (Figure S6B).

Several synaptic genes which are critical for neuronal communication, neurodevelopment, and cognitive development^46^ were also differentially up-regulated in EPN-E1 compared to EPN-E2, such as GRIN1, CHRNB1, CACNA1G, CACN1B, and P2RX5 (Figure S6C-G). EPN-E2 also had a distinct set of up-regulated synaptic markers such as GABRA5, DRD1, SCN4B, and P2RX7 (Figure S6H-K).

Further analysis revealed distinct pathway regulation between the EPN-E1 and EPN-E2 groups. EPN-E1 showed upregulation of Notch signaling, TP53, RAS, and interferon gamma (IFNG) pathways (Figure 6C, Figure S6L, Table S7A), as well as chromatin maintenance and G1/S-specific transcription. By contrast, EPN-E2 exhibited upregulation of hyaluronan biosynthesis, dopamine receptor signaling, voluntary skeletal muscle contraction, and antigen presentation pathways (Figure S6L, Table S7B). Single-cell RNA sequencing studies^21^^,^^22^ have shown that ependymoma samples contain ependymal-like and mesenchymal-like subpopulations. To assess mesenchymal character, we performed GSVA analysis. While some gene sets were more expressed in one cluster, there was no consistent mesenchymal expression pattern explaining the observed separation (Figure S6M).

### Projecting new patients onto a pre-existing UMAP reference landscape

Our established UMAP landscape has clearly delineated distinct biological regions corresponding to different disease types and subtypes. This landscape can also be leveraged to overlay new patients entering the clinic, aiding in the prediction of their disease subtype and ruling out misdiagnosis. To demonstrate this concept, we utilized previously developed algorithm^26^ to project new patient data onto the reference landscape.

One of the primary data sources for our landscape is the Children’s Brain Tumor Network (CBTN), which includes 77 ependymoma and 93 medulloblastoma samples among 23 different pediatric tumor types. Of the 93 medulloblastoma samples, 10 were classified as Group 3, 38 as Group 4, 24 as SHH, 9 as WNT, and 12 as NOS. We employed a nearest neighbors algorithm to overlay 12 NOS samples (A-L; Table S8, Figure 6D) onto our landscape. The results showed that patients H and I co-embedded within the EPN-E2 group, while patient F clustered with the WNT medulloblastomas. Patients A and G aligned with the MYC-amplified Group C2, and patient L corresponded with C1. Patient C localized to C4, whereas patients B and D were clearly situated within Group 4 (C6 and C5). Additionally, patients K, E, and J were positioned at the boundary between Group 3 and Group 4, within C5 and C3.

To validate the accuracy of our algorithm, we examined the molecular profiles of the 6 out of 12 NOS tumors that overlapped with the Group 3clusters. For instance, the median MYC expression value in C2 is 7.5 (log2(TPM)). Patients A, G, J, and L, all of whom fell within the MYC-amplified region, exhibited MYC expression levels of 6.56, 7.33, 7.28, and 6.25 (log2(TPM)), respectively (­Figure S6N). Additionally, patient A showed a gain of chromosome 8q, consistent with the profile of Group 3 subtypes. Patients C and K, located at the boundary between Group 3 and Group 4 tumors, also displayed elevated MYC expression (6.21 and 4.9, respectively), with patient K showing a gain of 8q.

Similarly, the Group 4 subclusters C6 and C5 are characterized by high EOMES expression, with a median expression value of 6.17 and 5.31 (log2(TPM)), respectively. Evaluating the 3 out of 12 NOS Patients B, D, and E that overlapped with Group 4 cluster, we observed that they exhibited elevated EOMES expression (6.37, 4.10, and 6.4, (log2(TPM)) respectively). Patient B also demonstrated a gain of 17q, aligning with the Group 4 subtype profile. We recalculated the UMAP with the 12 medulloblastoma NOS samples included and found that the predicted placement based on the nearest neighbors algorithm (shown in black) precisely matched the ground truth (red) in terms of their positioning on the landscape (Figure S6N).

Overlaying new patient data onto the landscape can also inform therapeutic decisions. For example, within the C2 group, characterized by high MYC expression, we found that stratifying patients based on MYC levels (Low = below the first quartile [Q1]; High = above the third quartile [Q3]; intermediate values excluded) revealed poor prognosis in patients with elevated MYC expression levels, as reported previously^47^ (*P* = .012, Figure 2E, Figure S6O). This subgroup also exhibited upregulation of MYC targets, EIF4EBP1, and pathways involved in translation (Figure 4F). EIF4EBP1 is a known negative regulator of translation initiation, and its elevated levels have been linked to drug resistance.^48^ Notably, EIF4EBP1 was similarly overexpressed in C2, mirroring the MYC expression pattern. When we further stratified patients based on EIF4EBP1 expression, those with high levels of EIF4EBP1 had poorer survival compared to those with lower expression (*P* = .081, Figure S6P).

Extending this analysis to pathway-level features, using a pre-specified threshold (High = GSVA > 0.6), Kaplan-Meier analyses showed worse overall survival for the High group for both HALLMARK_MYC_TARGETS_V2 (log-rank *P* < .0001; Figure G) and REACTOME_EUKARYOTIC_TRANSLATION_INITIATION (log-rank *P* = .0062; Figure I). In multivariable Cox models adjusting for age, sex, and cluster (C5 reference), High pathway activity remained independently associated with poorer survival (MYC targets: HR = 3.74, 95% CI 1.52-9.20, *P* = .004; Translation initiation: HR = 2.22, 95% CI 1.06-4.60, *P* = .033; Figure 6E and F, Figure S6Q). Cluster effects persisted, with C1 and C2 exhibiting elevated hazards relative to C5. Therefore, if a new patient, such as patient A (Figure 6D), falls into a region associated with high EIF4EBP1 expression, they may be at increased risk for drug resistance. Importantly, our landscape helps identify co-occurring transcriptional programs, such as Myc high expression and translation, that delineate highest-risk subgroups.

## Discussion

Over the past decade, the emergence of single-cell atlases^49^^,^^50^ and bulk RNASeq derived reference landscapes^10^^,^^26^ have emerged as powerful tools for molecular characterization of diseases. While scRNA-seq offers detailed insights into cellular heterogeneity, it requires significant time, resources, and complex analysis. In contrast, bulk RNA-seq landscapes constructed from publicly available datasets provide a cost-effective, scalable alternative, enabling rapid analysis of disease subtypes, molecular targets, and overall biology.

Our study demonstrates several key advantages of this approach. First, the scale and scope of our dataset revealed novel biological insights. We identified subtype-specific gene fusions and pathway regulation—such as increased translation in SHH, STAT5, and NTRK2 signaling in Group 4, and upregulation of TERT and WNT pathways in Group 3—offering potential therapeutic targets and improving disease classification.

Second, consistent with previous analysis,^33^ we identified two ependymoma clusters, EPN-E1 and EPN-E2. Each cluster showed subtype-specific fusions and pathway programs. Additionally, distinct enrichment of tyrosine receptor kinases and synaptic genes was also seen in EPN-E1 and EPN-E2, respectively—both of which are increasingly recognized as actionable targets in cancer. These findings suggest the existence of tumor-neuron synaptic signaling mechanisms in ependymomas, an area not yet explored.

Third, our landscape offers clinical utility. Mapping new patient samples to this reference allows inference of molecular features based on proximity to well-characterized neighbors—supporting diagnosis, clarifying ambiguous cases, and potentially guiding treatment. This is particularly valuable in atypical presentations or when traditional diagnostics are inconclusive. It also enables retrospective quality control, potentially identifying misclassified tumors.

Medulloblastoma, a well-characterized disease, served as a valuable internal control. In validating known findings, we confirmed the robustness of our approach. Studying medulloblastoma and ependymoma together also revealed ­unexpected transcriptional parallels between subtypes. For instance, *CHRNA4*, a nicotinic acetylcholine receptor gene implicated in epilepsy, was upregulated in both SHH medulloblastoma and EPN-E1. *GABRA5*, a GABA-A receptor subunit mediating inhibitory neurotransmission, was upregulated in MYC-amplified Group 3 and EPN-E2. These parallels suggest shared biological themes across tumor types and may highlight new avenues for pan-disease therapeutic exploration.

Finally, the publicly accessible landscape (via Oncoscape) serves as a valuable resource for hypothesis generation, biomarker discovery, and translational research. By offering an interactive tool, we enable the broader scientific community to explore and analyze pediatric CNS tumors with greater flexibility and depth.

In conclusion, bulk RNA-seq-based reference landscapes represent a scalable, informative, and clinically relevant tool for disease stratification and discovery. Their integration into research and clinical frameworks has the potential to accelerate precision diagnostics and therapeutic development across pediatric neuro-oncology.

## Supplementary Material

noaf251_Supplementary_Data

## Data Availability

The datasets were dervived from sources in public domain in E-MTAB-6814, GSE109381, EGAS00001002696, EGAS00001000254, EGAD00001006305, and EGAS00001005826. All custom code used in this study are available at https://github.com/sonali-bioc/MedulloEPNLandscape. All analysis including statistics and visualization were done in R version 4.3. Plots were generated using R basic graphics and ggplot2.
