# Supplementary material for: Integrated transcriptomic landscape of medulloblastoma and ependymoma reveals novel tumor subtype-specific biology
Source: Neuro Oncol. 2025 Oct 24;28(2):505–19. doi: 10.1093/neuonc/noaf251 (PMC12979040; doi:10.1093/neuonc/noaf251)
Supplement: noaf251_Supplementary_Data [file noaf251_supplementary_data.zip › noaf251_Supplementary_Data/Supplementary Table and Figure Legends_10_13_2025.docx]

**Supplemental Figures**

- **Fig S1. Dimension Reduction and Normalization Techniques Applied to RNA-seq Data** (A-C) Dimension reduction techniques applied to non-batch corrected log2(TPM+1) data: (A) PCA, (B) t-SNE, (C) UMAP. (D-F) Dimension reduction techniques applied to batch-corrected log2(TPM+1) data: (D) PCA, (E) t-SNE, (F) UMAP. (G-I) Different normalization methods applied to batch-corrected data: (G) CPM normalization, (H) RPKM normalization, (I) VST normalization. (J) Stacked barplots showing proportion of samples with amplifications and deletions in chr arm 17p, 17q, 1q, 22q, 9p, 9q (K) Boxplots showing gene expression ( log2(TPM+1)) of genes : ATOH1, MYC, KCNA1, WNT5A , TGFB1 and IGF2.
- **Fig S2. Further Validation of the Reference Landscape and Subtypes of Medulloblastoma and Ependymoma** (A-F) Validation based on gene expression patterns across different medulloblastoma and ependymoma subtypes. SFRP1 and HHIP show elevated expression of SHH medulloblastoma, GABRA5 and IMPG2 show elevlates expression in Group3 medulloblastoma whereas EOMES and RBM24 show elevated gene expression in Group4 medulloblastoma patients. (G-K) Validation based on gene fusions identified in the different medulloblastoma subtypes, as previously shown by Luo et al^23^. (L-Q) Genes showing elevated gene expression in posterior fossa((L-N) and supratentorial (O-Q) ependymomas.
- **Fig S3. Copy Number analysis for various medulloblastoma clusters** (A) Survival analysis for S2 based on the copy number profile of 9q. (B) Manhattan plot showing the percentage of tumors with amplifications and deletions in each of the 3 subclusters of Shh medulloblastoma - S1, S2 and S3 and(C) six clusters of group3 and group4 medulloblastoma. (D=E) Kaplan-Meier plots for C4 based on gender and copy number status of 4p (Male(blue) vs Female(pink), p=0.015, n[Male]=18, n[Female]=6, Copy No deletions(green) vs Copy no neutral(grey), pvalue =0.032, n[Copy no deletions]=10, n[copy no neutral] =14).
- **Fig S4. Clustering Methods Validate Group 3 and Group 4 Medulloblastoma Clusters** (A-I) GSVA scores for pathways regulated in Group 3 and Group 4 medulloblastoma, visualized on the UMAP. (J) UMAP showing scRNASeq data from 25 patients colored in subtype (K) Dotplot showing gene expression of genes (x-axis) in each of subtypes for scRNASeq data (L) ViolinPlots showing gene expression of genes (x-axis) in each of subtypes for scRNASeq data (M) Heatmap showing the average pathway score for pathways shown in Fig4, for each subtype of scRNASeq data.
- **Fig S5. Clustering Methods Validate EPN-E1 and EPN-E2** Tumor purity estimates based on (A) PurEE and (B) ESTIMATE. (C) Heatmap displaying the average expression of the 8,000 most variable genes across forebrain and hindbrain samples, as well as EPN-E1 and EPN-E2 samples. (D) Correlation plot illustrating relationships among forebrain and hindbrain samples and EPN-E1 and EPN-E2, based on the 8,000 most variable genes.
- **Fig S6. Differential gene expression analysis for EPN-E1 and EPN-E2** (A) Volcano plot showing DEGs upregulated in E1 containing ZFTA:RELA gene fusions vs E2 containing ZFTA:RELA gene fusions. (b) Volcano plot showing DEGs upregulated within E1 containing ZFTA:RELA vs those that did not contain ZFTA:RELA gene fusion (C-K) Synaptic genes upregulated in EPN -E1 and EPN-e2 respectively (l) UMAP showing GSVA scores for pathways regulated in EPN-E1 and EPN-E2 (m) Heatmap showing average of mesenchymal scores across EPN-E1 and EPN-E2. (n)Validation of a new patient overlayed on the reference landscape, based on gene expression and copy number patterns.(o) Kaplan Meier plots for C2 based on MYC gene expression levels (High(red) vs Low(orange), pvalue = 0.012, n[high exp]=35, n[low exp]=109 ). (p) Kaplan Meier plots for C2 based on EIF4EBP1 gene expression levels (High(red) vs Low,(orange) pvalue = 0.0081, n[high exp]=14, n[low exp]=77 ). (q) Kaplan–Meier curves for REACTOME_EUKARYOTIC_TRANSLATION_INITIATION and dichotomized at the pre-specified GSVA cutoff (High = GSVA > 0.6, Low = GSVA ≤ 0.6). Log-rank p-values are shown

**Supplemental Tables**

- **Table S1**. Datasets from North America and Europe were combined to generate the medulloblastoma UMAP
- **Table2 S2** Copy number profiles for each subtype of Group3 and Group4 medulloblastoma , and each subtype of shh medulloblastoma, as obtained from consensus clustering.
- **Table S3** Top recurrent RNA gene fusions in ST-EPNs in EPN-E1
- **Table S4** Top recurrent RNA gene fusions in PF-EPNs in EPN-E2
- **Table S5** Top recurrent RNA fusions in medulloblastoma subtypes
- **Table S6** All genes and kinases upregulated in EPN-E1 and EPN=E2
- **Table S7** Pathways upregulated in EPN-E1 and EPN-E2
- **Table S8** Mapping of 12 NOS medulloblastoma samples from CBTN as shown in Figure 6
