# Supplementary material for: Integrated transcriptomic landscape of medulloblastoma and ependymoma reveals novel tumor subtype-specific biology
Source: Neuro Oncol. 2025 Oct 24;28(2):505–19. doi: 10.1093/neuonc/noaf251 (PMC12979040; doi:10.1093/neuonc/noaf251)
Supplement: noaf251_Supplementary_Data [file noaf251_supplementary_data.zip › noaf251_Supplementary_Data/supp_figs_10_13_2025.pdf]

Integrated transcriptomic landscape of medulloblastoma and ependymoma reveals novel tumor subtype-specific biology

Sonali Arora[1], Nicholas Nuechterlein[2], Matt Jensen[1], Gregory Glatzer[1], Philipp Sievers[3], Srinidhi Varadharajan[4], Andrey Korshunov[3], Felix Sahm[3], Stephen C. Mack[4], Michael D. Taylor[5], Taran Gujral[1], Eric C Holland*[1].

Supplementary Information

Table of contents

Supplementary Figures S1-S7

Supplementary Figure Legends

Suppl Figure legends

- **Fig S1. Dimension Reduction and Normalization Techniques Applied to RNA-seq Data** (A-C) Dimension reduction techniques applied to non-batch corrected log2(TPM+1) data: (A) PCA, (B) t-SNE, (C) UMAP. (D-F) Dimension reduction techniques applied to batch-corrected log2(TPM+1) data: (D) PCA, (E) t-SNE, (F) UMAP. (G-I) Different normalization methods applied to batch-corrected data: (G) CPM normalization, (H) RPKM normalization, (I) VST normalization. (J) Stacked barplots showing proportion of samples with amplifications and deletions in chr arm 17p, 17q, 1q, 22q, 9p, 9q (K) Boxplots showing gene expression (log2(TPM+1)) of genes : ATOH1, MYC, KCNA1, WNT5A , TGFB1 and IGF2.
- **Fig S2. Further Validation of the Reference Landscape and Subtypes of Medulloblastoma and Ependymoma** (A-F) Validation based on gene expression patterns across different medulloblastoma and ependymoma subtypes. SFRP1 and HHIP show elevated expression of SHH medulloblastoma, GABRA5 and IMPG2 show elevlates expression in Group3 medulloblastoma whereas EOMES and RBM24 show elevated gene expression in Group4 medulloblastoma patients. (G-K) Validation based on gene fusions identified in the different medulloblastoma subtypes, as previously shown by Luo et al[23]. Genes showing elevated gene expression in posterior fossa((L-N) and supratentorial (O-Q) ependymomas.
- **Fig S3. Copy Number analysis for various medulloblastoma clusters** (A) Survival analysis for S2 based on the copy number profile of 9q. (B) Manhattan plot showing the percentage of tumors with amplifications and deletions in each of the 3 subclusters of Shh medulloblastoma - S1, S2 and S3 and(C) six clusters of group3 and group4 medulloblastoma. (D=E) Kaplan-Meier plots for C4 based on gender and copy number status of 4p (Male(blue) vs Female(pink), p=0.015, n[Male]=18, n[Female]=6, Copy No deletions(green) vs Copy no neutral(grey), pvalue =0.032, n[Copy no deletions]=10, n[copy no neutral] =14).
- **Fig S4. Clustering Methods Validate Group 3 and Group 4 Medulloblastoma Clusters** (A-I) GSVA scores for pathways regulated in Group 3 and Group 4 medulloblastoma, visualized on the UMAP. (J) UMAP showing scRNASeq data from 25 patients colored in subtype (K) Dotplot showing gene expression of genes (x-axis) in each of subtypes for scRNASeq data (L) ViolinPlots showing gene expression of genes (x-axis) in each of subtypes for scRNASeq data (M) Heatmap showing the average pathway score for pathways shown in Fig4, for each subtype of scRNASeq data.
- **Fig S5. Clustering Methods Validate EPN-E1 and EPN-E2**
- Tumor purity estimates based on (A) PurEE and (B) ESTIMATE. (C) Heatmap displaying the average expression of the 8,000 most variable genes across forebrain and hindbrain samples, as well as EPN-E1 and EPN-E2 samples. (D) Correlation plot illustrating relationships among forebrain and hindbrain samples and EPN-E1 and EPN-E2, based on the 8,000 most variable genes.
- **Fig S6. Differential gene expression analysis for EPN-E1 and EPN-E2** (A) Volcano plot showing DEGs upregulated in E1 containing ZFTA:RELA gene fusions vs E2 containing ZFTA:RELA gene fusions. (b) Volcano plot showing DEGs upregulated within E1 containing ZFTA:RELA vs those that did not contain ZFTA:RELA gene fusion (C-K) Synaptic genes upregulated in EPN - E1 and EPN-e2 respectively (l) UMAP showing GSVA scores for pathways regulated in EPN-E1 and EPN-E2 (m) Heatmap showing average of mesenchymal scores across EPN-E1 and EPN-E2.
- **Fig S7. Validation of New Patient Data Overlay on the Reference Landscape (A)**Validation of a new patient overlayed on the reference landscape, based on gene expression and copy number patterns.(B) Kaplan Meier plots for C2 based on MYC gene expression levels (High(red) vs Low(orange), pvalue = 0.012, n[high exp]=35, n[low exp]=109). (C) Kaplan Meier plots for C2 based on EIF4EBP1 gene expression levels (High(red) vs Low,(orange) pvalue = 0.0081, n[high exp]=14, n[low exp]=77).

Supp Fig 1

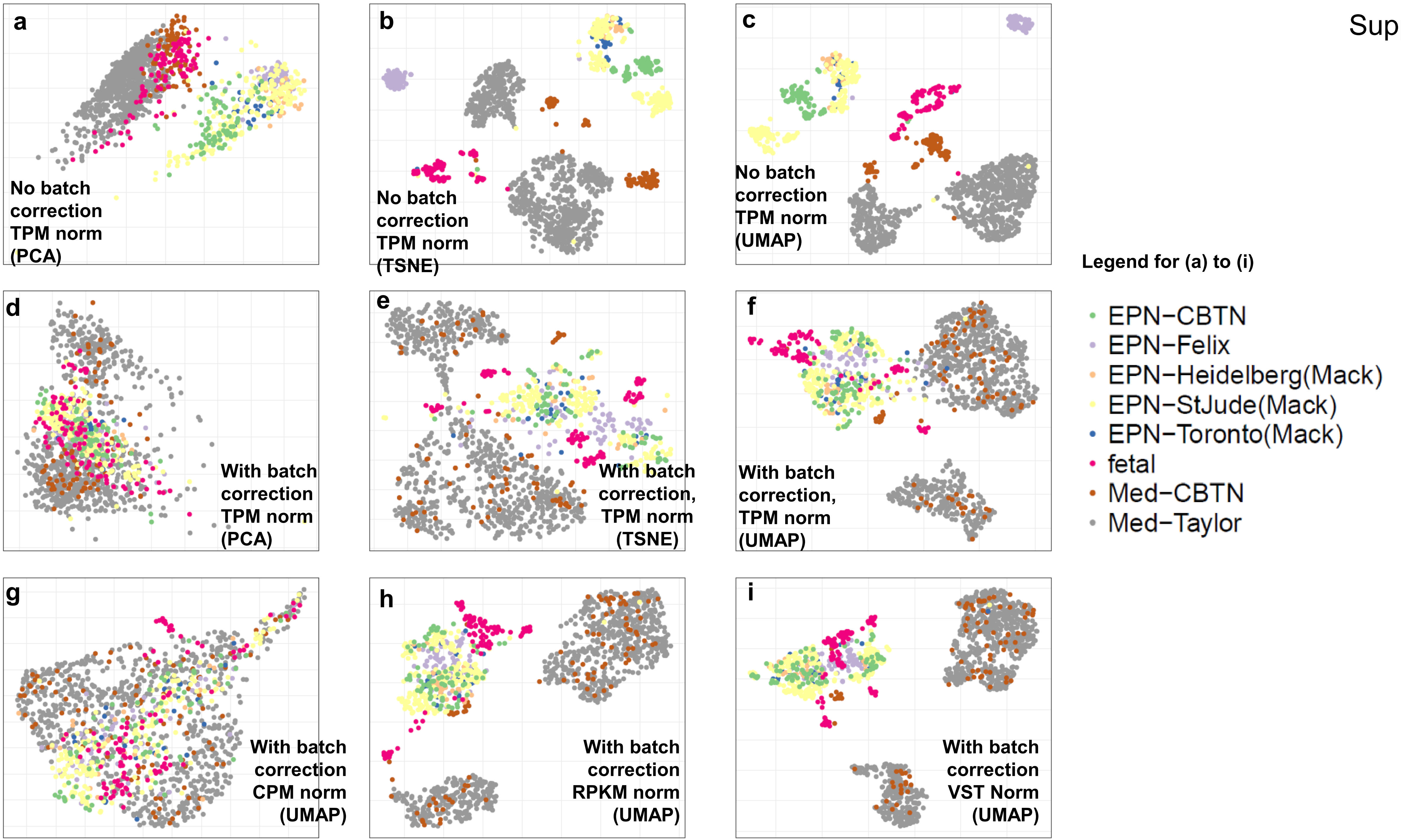
a
No batch correction TPM norm (PCA)
b
No batch correction TPM norm (TSNE)
c
No batch correction TPM norm (UMAP)
d
With batch correction TPM norm (PCA)
e
With batch correction, TPM norm (TSNE)
f
With batch correction, TPM norm (UMAP)
g
With batch correction CPM norm (UMAP)
h
With batch correction RPKM norm (UMAP)
i
With batch correction VST Norm (UMAP)
Legend for (a) to (i)
EPN−CBTN
EPN−Felix
EPN−Heidelberg(Mack)
EPN−StJude(Mack)
EPN−Toronto(Mack)
fetal
Med−CBTN
Med−Taylor

j

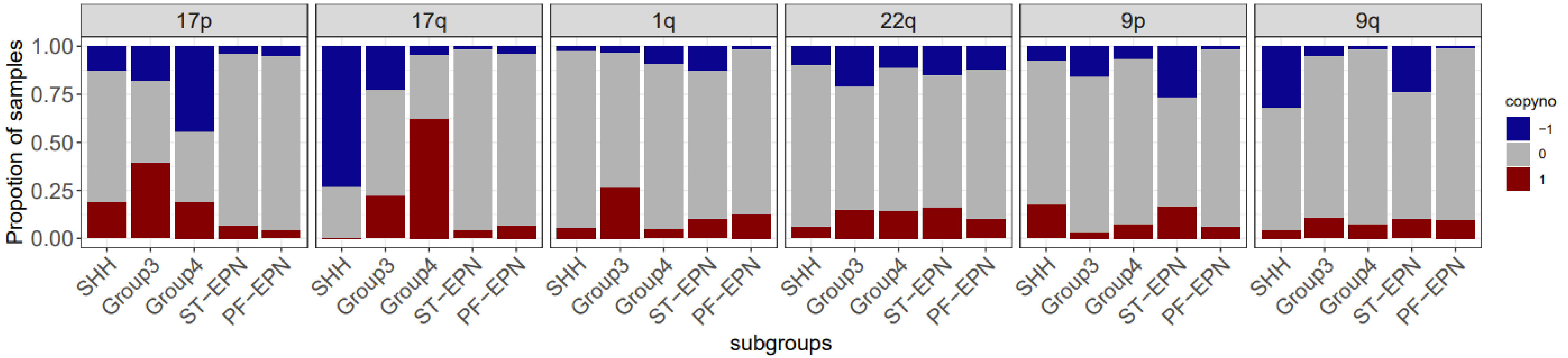
17p
17q
1q
22q
9p
9q
Propotion of samples
1.00
0.75
0.50
0.25
0.00
SHH
Group3
Group4
ST−EPN
PF−EPN
subgroups
copyno
−1
0
1

k

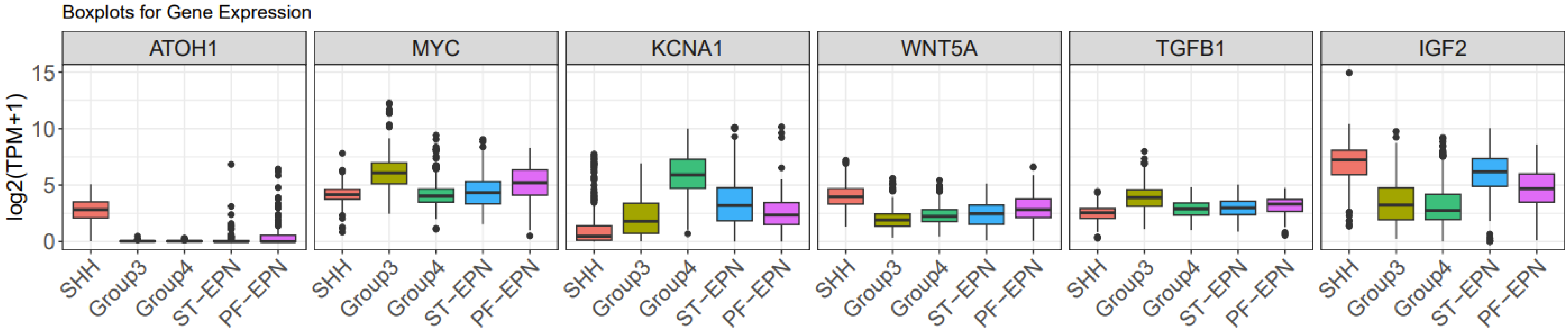
Boxplots for Gene Expression
ATOH1
MYC
KCNA1
WNT5A
TGFB1
IGF2
log2(TPM+1)
15
10
5
0
SHH
Group3
Group4
ST−EPN
PF−EPN

Supp Fig 2

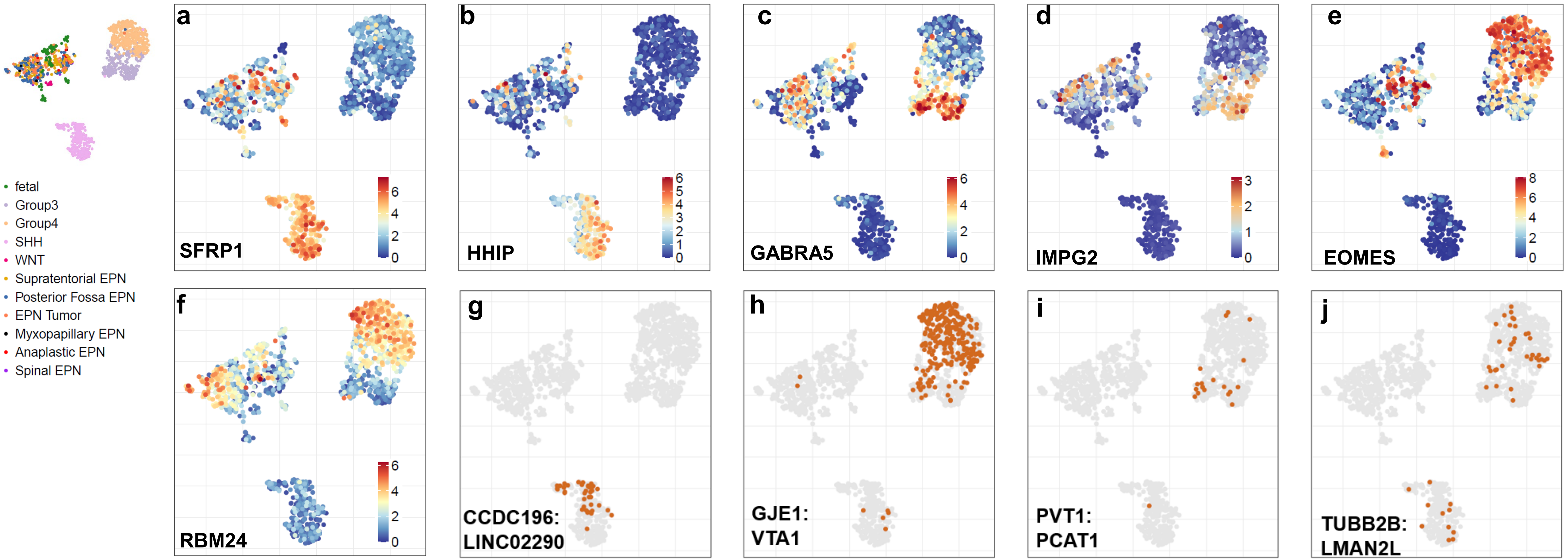

fetal
Group3
Group4
SHH
WNT
Supratentorial EPN
Posterior Fossa EPN
EPN Tumor
Myxopapillary EPN
Anaplastic EPN
Spinal EPN
a
SFRP1
b
HHIP
c
GABRA5
d
IMPG2
e
EOMES
f
RBM24
g
CCDC196:
LINC02290
h
GJE1:
VTA1
i
PVT1:
PCAT1
j
TUBB2B:
LMAN2L

Supp Fig 2

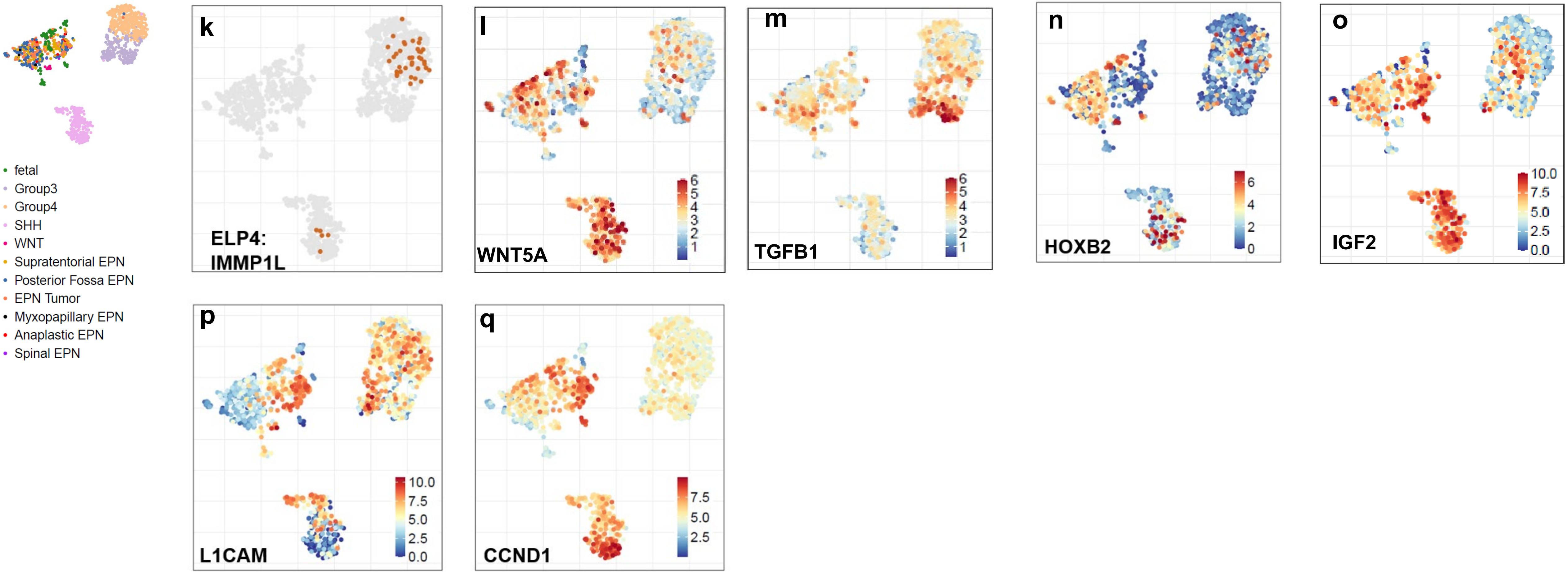

fetal
Group3
Group4
SHH
WNT
Supratentorial EPN
Posterior Fossa EPN
EPN Tumor
Myxopapillary EPN
Anaplastic EPN
Spinal EPN
k
ELP4:
IMMP1L
l
WNT5A
m
TGFB1
n
HOXB2
o
IGF2
p
L1CAM
q
CCND1

Supp Fig 3

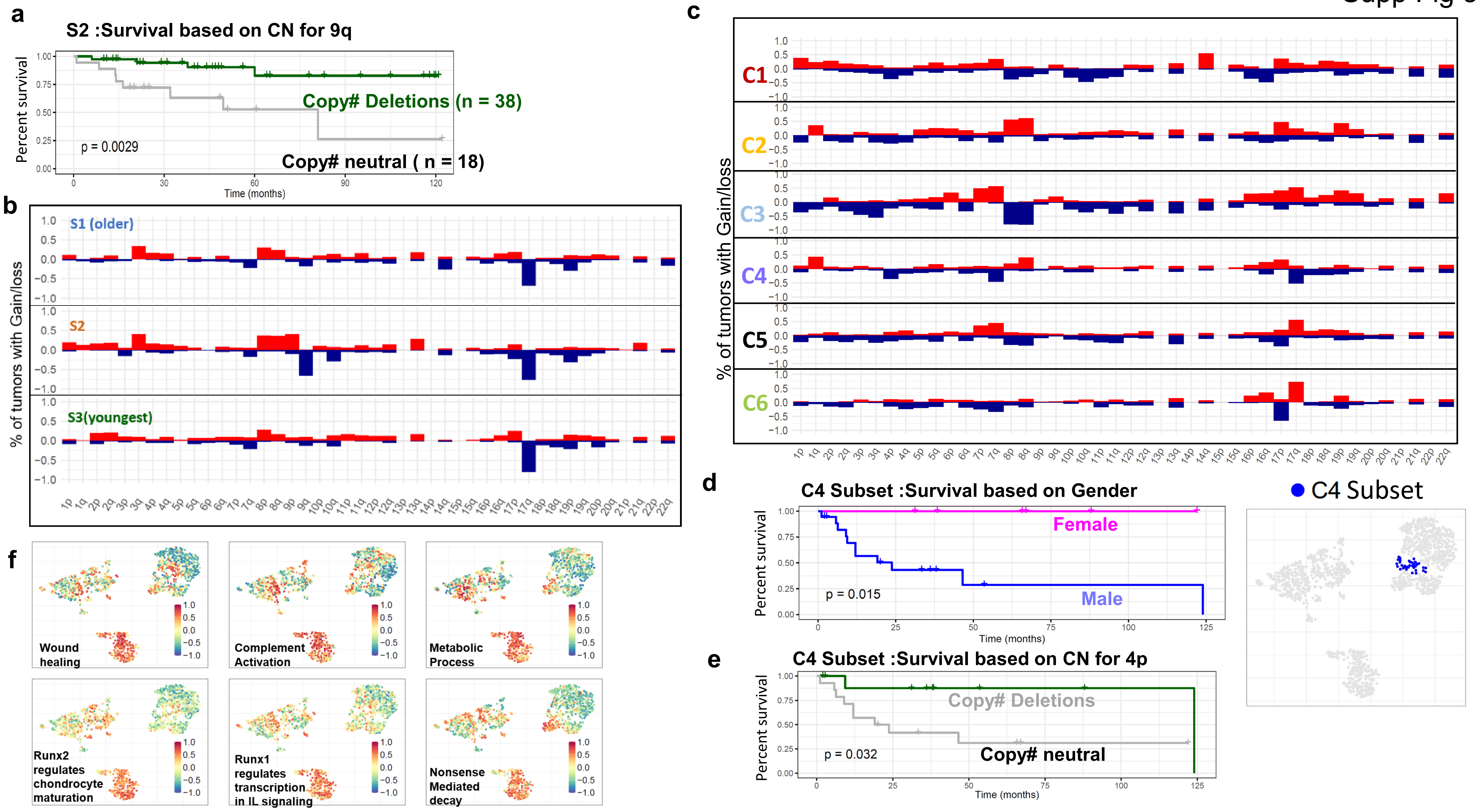

a
S2 :Survival based on CN for 9q
Percent survival
Copy# Deletions (n = 38)
Copy# neutral (n = 18)
p = 0.0029
Time (months)
b
% of tumors with Gain/loss
S1 (older)
S2
S3(youngest)
c
C1
C2
C3
C4
C5
C6
d
C4 Subset :Survival based on Gender
Female
Male
p = 0.015
C4 Subset
e
C4 Subset :Survival based on CN for 4p
Copy# Deletions
Copy# neutral
p = 0.032
f
Wound healing
Complement Activation
Metabolic Process
Runx2 regulates chondrocyte maturation
Runx1 regulates transcription in IL signaling
Nonsense Mediated decay

Supp Fig 4

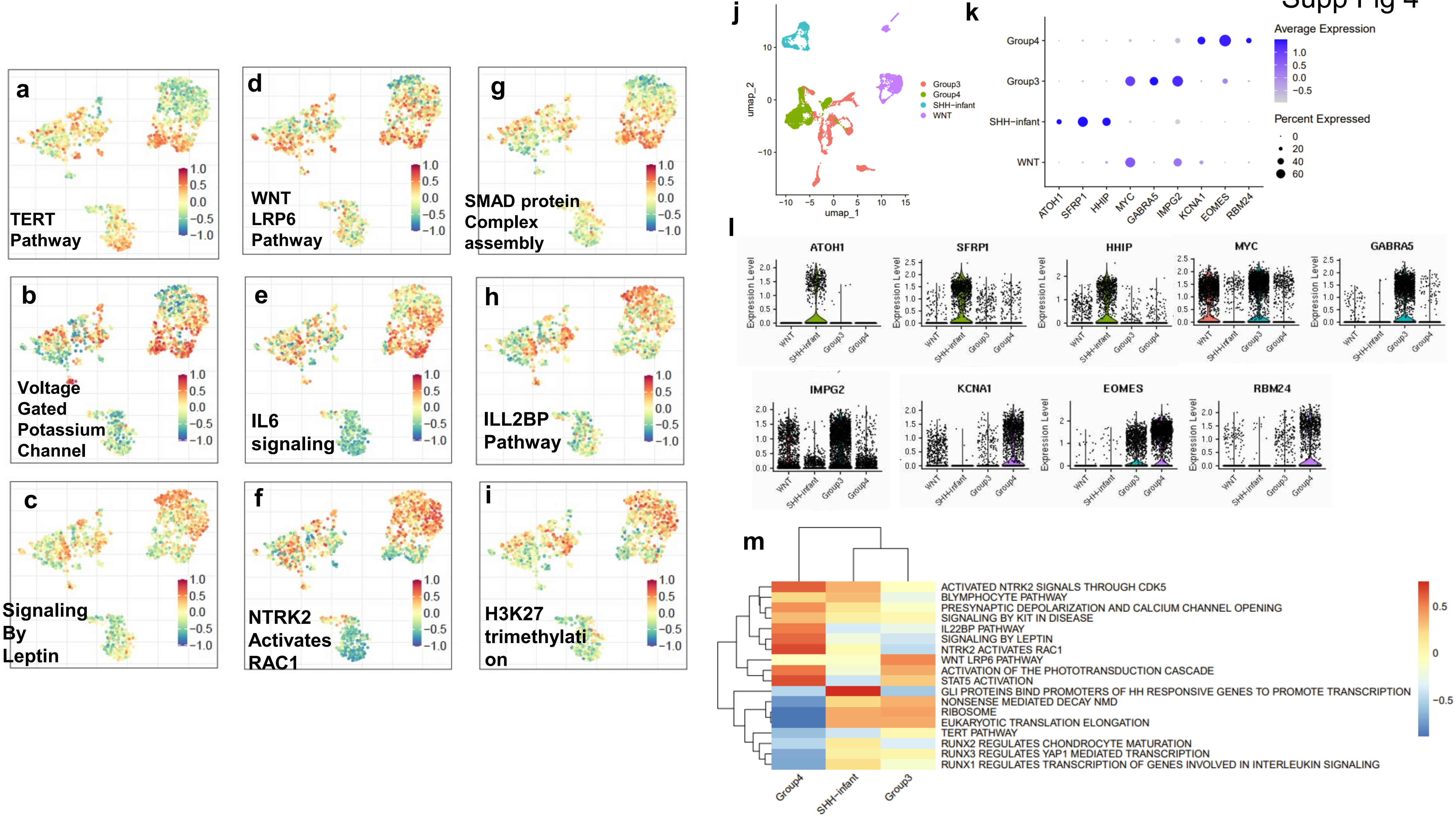
a
TERT Pathway
d
WNT LRP6 Pathway
g
SMAD protein Complex assembly
b
Voltage Gated Potassium Channel
e
IL6 signaling
h
ILL2BP Pathway
c
Signaling By Leptin
f
NTRK2 Activates RAC1
i
H3K27 trimethylati on
j
umap_1
umap_2
Group3
Group4
SHH-infant
WNT
k
Average Expression
Percent Expressed
Group4
Group3
SHH−infant
WNT
ATOH1
SFRP1
HHIP
MYC
GABRA5
IMPG2
KCNA1
EOMES
RBM24
l
Expression Level
m
ACTIVATED NTRK2 SIGNALS THROUGH CDK5
BLYMPHOCYTE PATHWAY
PRESYNAPTIC DEPOLARIZATION AND CALCIUM CHANNEL OPENING
SIGNALING BY KIT IN DISEASE
IL22BP PATHWAY
SIGNALING BY LEPTIN
NTRK2 ACTIVATES RAC1
WNT LRP6 PATHWAY
ACTIVATION OF THE PHOTOTRANSDUCTION CASCADE
STAT5 ACTIVATION
GLI PROTEINS BIND PROMOTERS OF HH RESPONSIVE GENES TO PROMOTE TRANSCRIPTION
NONSENSE MEDIATED DECAY NMD
RIBOSOME
EUKARYOTIC TRANSLATION ELONGATION
TERT PATHWAY
RUNX2 REGULATES CHONDROCYTE MATURATION
RUNX3 REGULATES YAP1 MEDIATED TRANSCRIPTION
RUNX1 REGULATES TRANSCRIPTION OF GENES INVOLVED IN INTERLEUKIN SIGNALING
Group4
SHH-infant
Group3

Supp Fig 5

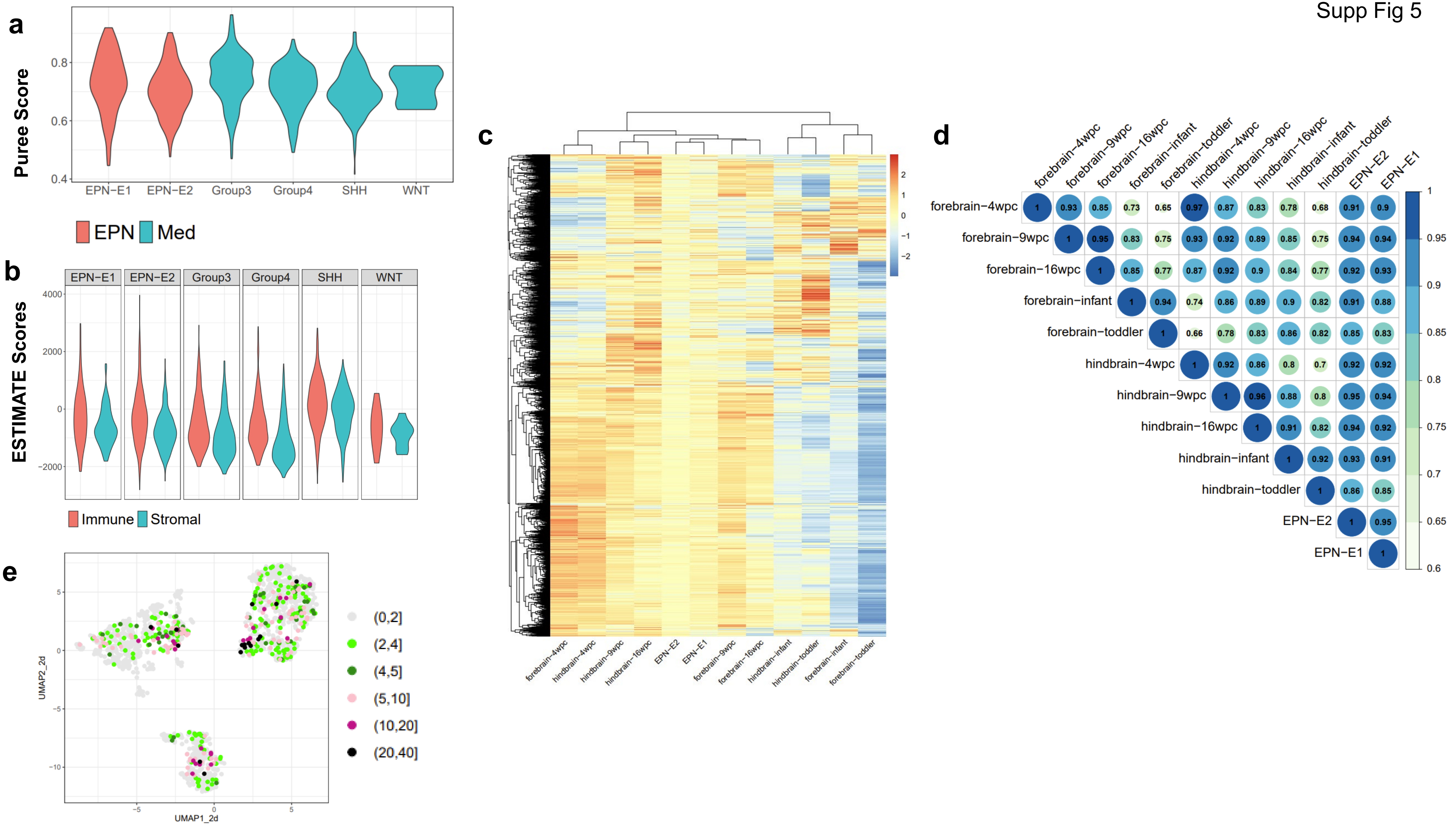
a
Puree Score
0.4
0.6
0.8
EPN−E1
EPN−E2
Group3
Group4
SHH
WNT
EPN
Med
b
ESTIMATE Scores
EPN−E1
EPN−E2
Group3
Group4
SHH
WNT
4000
2000
0
−2000
Immune
Stromal
c
forebrain−4wpc
hindbrain−4wpc
hindbrain−9wpc
hindbrain−16wpc
EPN−E2
EPN−E1
forebrain−9wpc
forebrain−16wpc
hindbrain−infant
hindbrain−toddler
forebrain−infant
forebrain−toddler
2
1
0
−1
−2
d
forebrain−4wpc
forebrain−9wpc
forebrain−16wpc
forebrain−infant
forebrain−toddler
hindbrain−4wpc
hindbrain−9wpc
hindbrain−16wpc
hindbrain−infant
hindbrain−toddler
EPN−E2
EPN−E1
1
0.95
0.9
0.85
0.8
0.75
0.7
0.65
0.6
e
UMAP1_2d
UMAP2_2d
(0,2]
(2,4]
(4,5]
(5,10]
(10,20]
(20,40]

Supp Fig 6

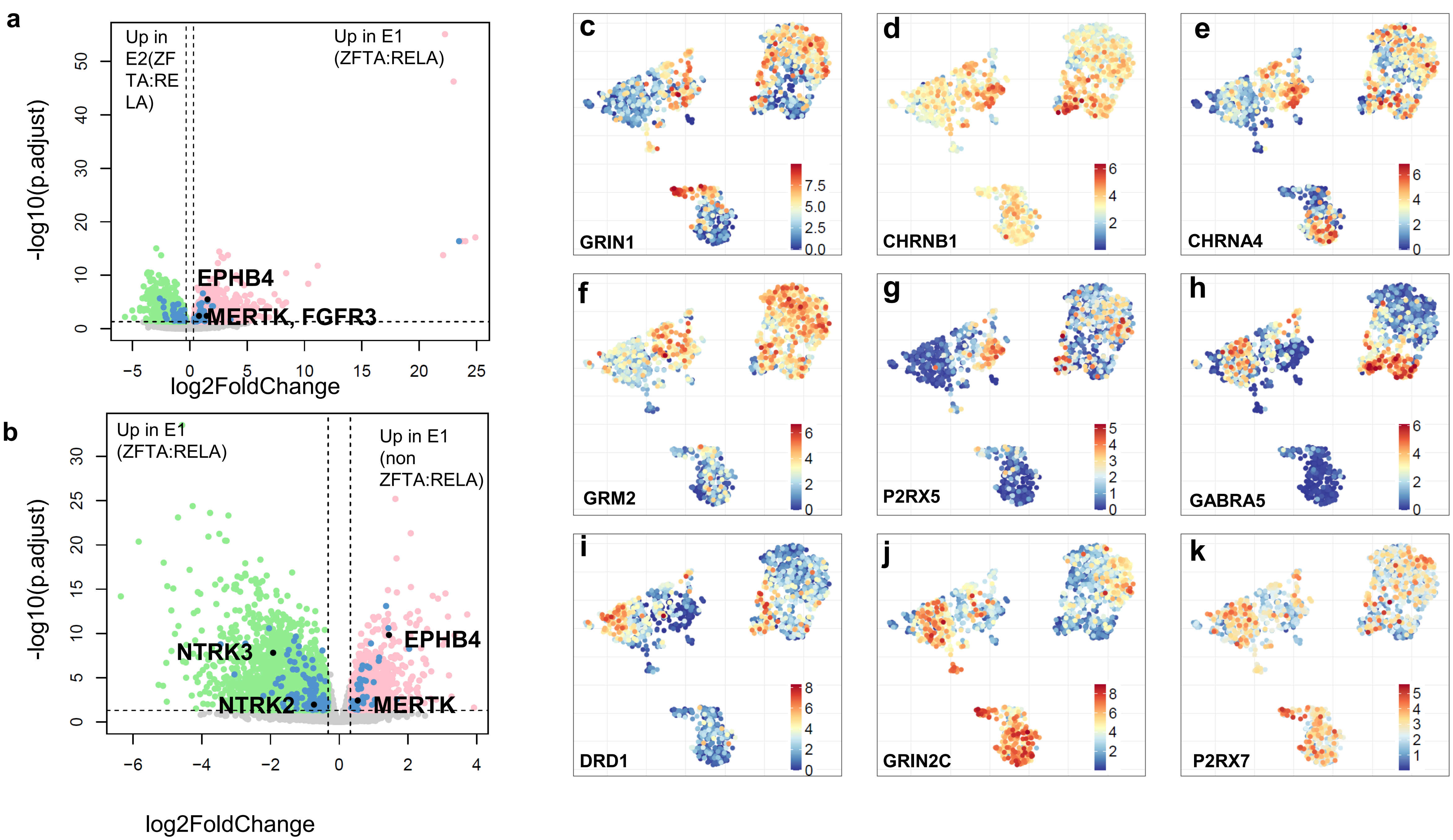
a
Up in E2(ZFTA:RELA)
Up in E1 (ZFTA:RELA)
EPHB4
MERTK, FGFR3
-log10(p.adjust)
log2FoldChange
b
Up in E1 (ZFTA:RELA)
Up in E1 (non ZFTA:RELA)
NTRK3
NTRK2
EPHB4
MERTK
-log10(p.adjust)
log2FoldChange
c
GRIN1
d
CHRNB1
e
CHRNA4
f
GRM2
g
P2RX5
h
GABRA5
i
DRD1
j
GRIN2C
k
P2RX7

Supp Fig 6

l

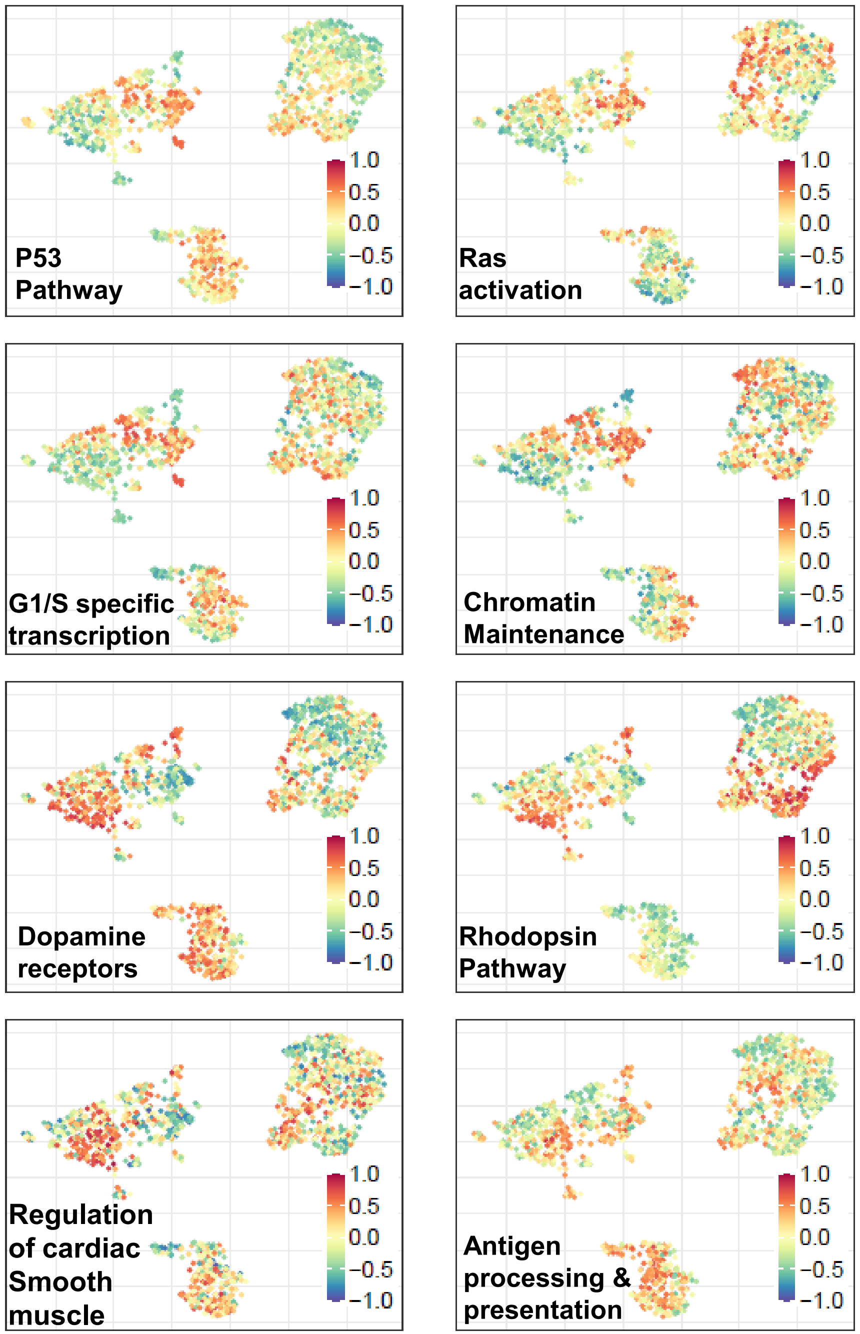

P53 Pathway
Ras activation
G1/S specific transcription
Chromatin Maintenance
Dopamine receptors
Rhodopsin Pathway
Regulation of cardiac Smooth muscle
Antigen processing & presentation
1.0
0.5
0.0
−0.5
−1.0

m

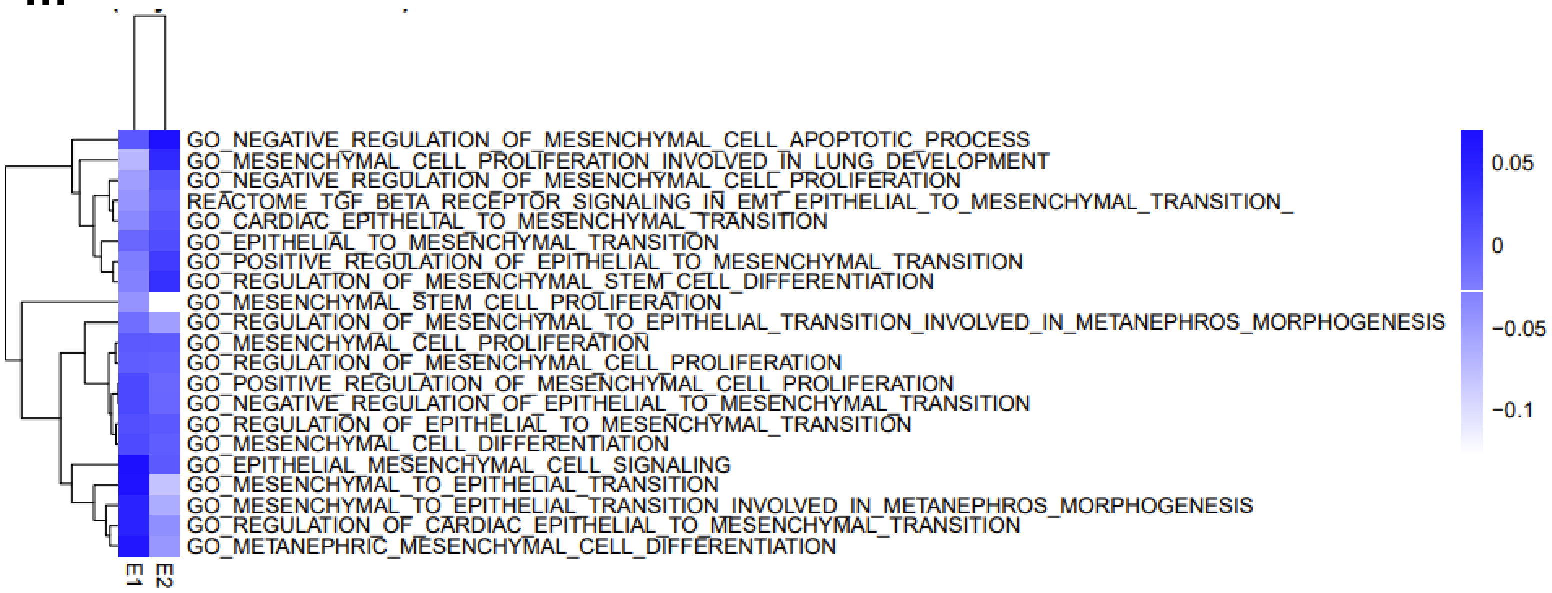

GO_NEGATIVE_REGULATION_OF_MESENCHYMAL_CELL_APOPTOTIC_PROCESS
GO_MESENCHYMAL_CELL_PROLIFERATION_INVOLVED_IN_LUNG_DEVELOPMENT
GO_NEGATIVE_REGULATION_OF_MESENCHYMAL_CELL_PROLIFERATION
REACTOME_TGF_BETA_RECEPTOR_SIGNALING_IN_EMT_EPITHELIAL_TO_MESENCHYMAL_TRANSITION_
GO_CARDIAC_EPITHELIAL_TO_MESENCHYMAL_TRANSITION
GO_EPITHELIAL_TO_MESENCHYMAL_TRANSITION
GO_POSITIVE_REGULATION_OF_EPITHELIAL_TO_MESENCHYMAL_TRANSITION
GO_REGULATION_OF_MESENCHYMAL_STEM_CELL_DIFFERENTIATION
GO_MESENCHYMAL_STEM_CELL_PROLIFERATION
GO_REGULATION_OF_MESENCHYMAL_TO_EPITHELIAL_TRANSITION_INVOLVED_IN_METANEPHROS_MORPHOGENESIS
GO_MESENCHYMAL_CELL_PROLIFERATION
GO_REGULATION_OF_MESENCHYMAL_CELL_PROLIFERATION
GO_POSITIVE_REGULATION_OF_MESENCHYMAL_CELL_PROLIFERATION
GO_NEGATIVE_REGULATION_OF_EPITHELIAL_TO_MESENCHYMAL_TRANSITION
GO_REGULATION_OF_EPITHELIAL_TO_MESENCHYMAL_TRANSITION
GO_MESENCHYMAL_CELL_DIFFERENTIATION
GO_EPITHELIAL_MESENCHYMAL_CELL_SIGNALING
GO_MESENCHYMAL_TO_EPITHELIAL_TRANSITION
GO_MESENCHYMAL_TO_EPITHELIAL_TRANSITION_INVOLVED_IN_METANEPHROS_MORPHOGENESIS
GO_REGULATION_OF_CARDIAC_EPITHELIAL_TO_MESENCHYMAL_TRANSITION
GO_METANEPHRIC_MESENCHYMAL_CELL_DIFFERENTIATION
E1
E2
0.05
0
−0.05
−0.1

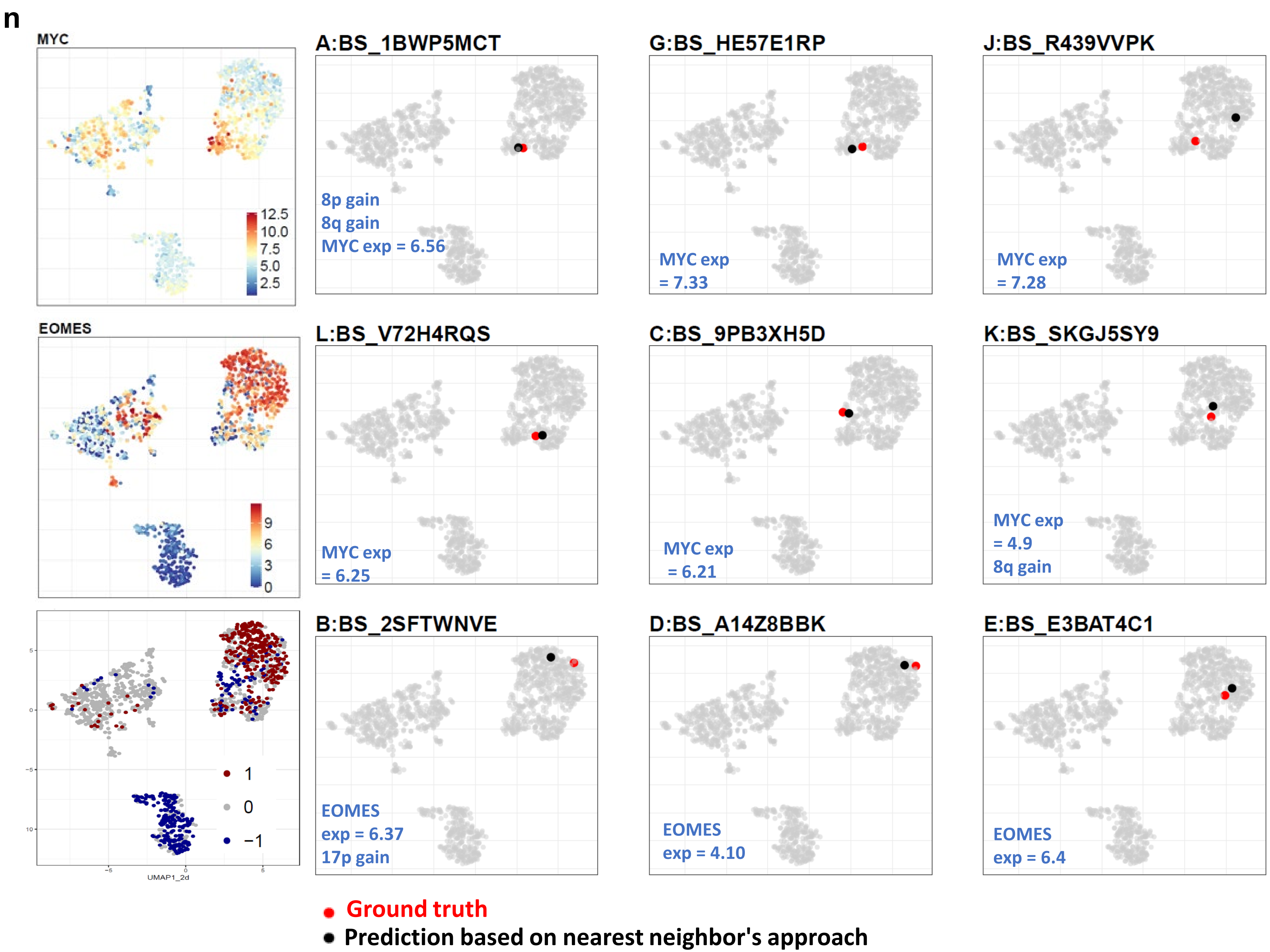
n
MYC
12.5
10.0
7.5
5.0
2.5
EOMES
9
6
3
0
1
0
−1
UMAP1_2d
A:BS_1BWP5MCT
8p gain
8q gain
MYC exp = 6.56
G:BS_HE57E1RP
MYC exp
= 7.33
J:BS_R439VVPK
MYC exp
= 7.28
L:BS_V72H4RQS
MYC exp
= 6.25
C:BS_9PB3XH5D
MYC exp
= 6.21
K:BS_SKGJ5SY9
MYC exp
= 4.9
8q gain
B:BS_2SFTWNVE
EOMES
exp = 6.37
17p gain
D:BS_A14Z8BBK
EOMES
exp = 4.10
E:BS_E3BAT4C1
EOMES
exp = 6.4
Ground truth
Prediction based on nearest neighbor's approach

Supp Fig 6

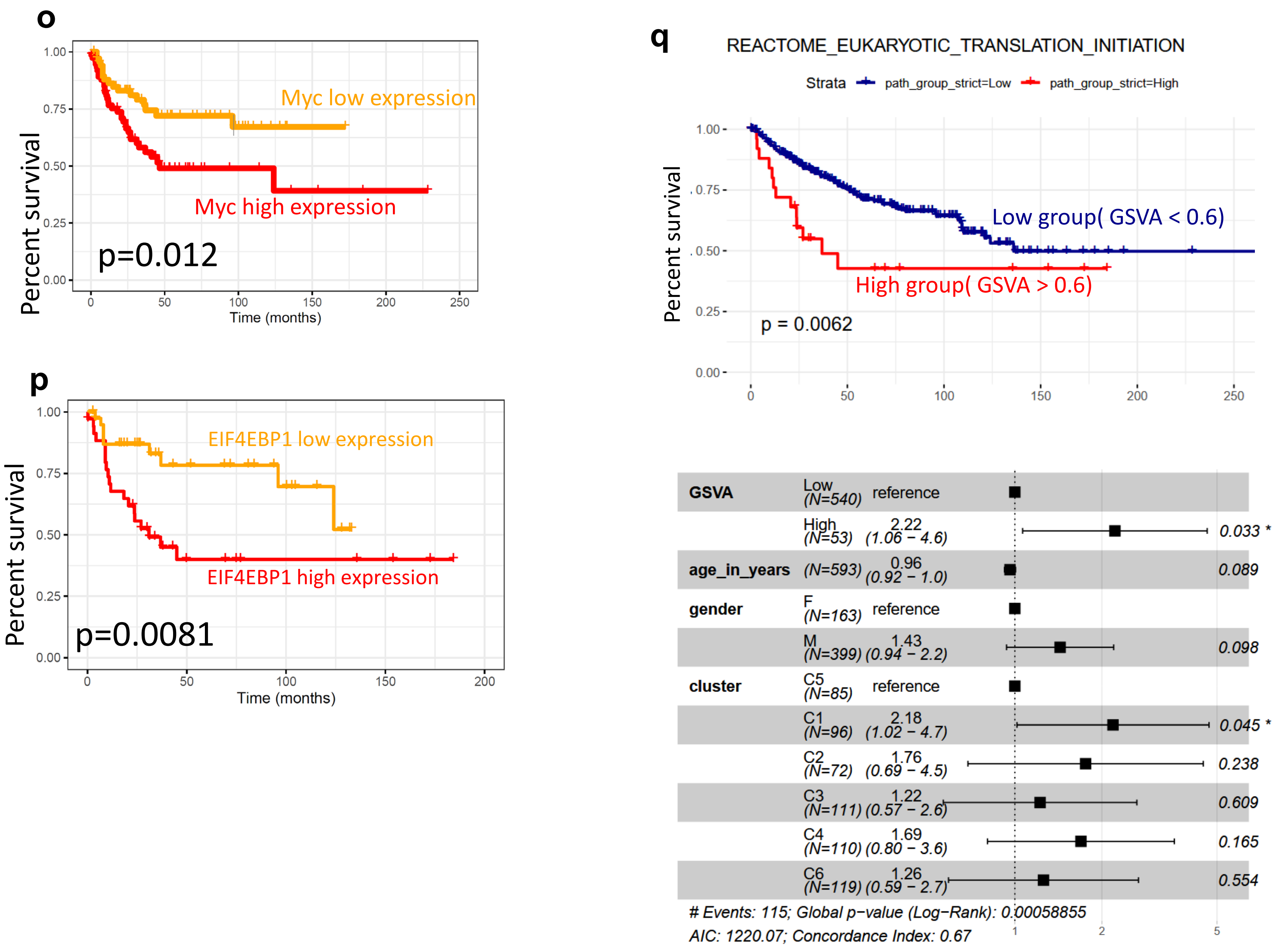
o
Percent survival
1.00
0.75
0.50
0.25
0.00
0
50
100
150
200
250
Time (months)
Myc low expression
Myc high expression
p=0.012
p
Percent survival
EIF4EBP1 low expression
EIF4EBP1 high expression
p=0.0081
Time (months)
q
REACTOME_EUKARYOTIC_TRANSLATION_INITIATION
Strata
path_group_strict=Low
path_group_strict=High
Percent survival
Low group(GSVA < 0.6)
High group(GSVA > 0.6)
p = 0.0062
GSVA
Low (N=540) reference
High (N=53) 2.22 (1.06 − 4.6) 0.033 *
age_in_years (N=593) 0.96 (0.92 − 1.0) 0.089
gender
F (N=163) reference
M (N=399) 1.43 (0.94 − 2.2) 0.098
cluster
C5 (N=85) reference
C1 (N=96) 2.18 (1.02 − 4.7) 0.045 *
C2 (N=72) 1.76 (0.69 − 4.5) 0.238
C3 (N=111) 1.22 (0.57 − 2.6) 0.609
C4 (N=110) 1.69 (0.80 − 3.6) 0.165
C6 (N=119) 1.26 (0.59 − 2.7) 0.554
Events: 115; Global p−value (Log−Rank): 0.00058855
AIC: 1220.07; Concordance Index: 0.67
1
2
5
